# The acylfulvene alkylating agent, LP-184, retains nanomolar potency in non-small cell lung cancer carrying otherwise therapy-refractory mutations

**DOI:** 10.18632/oncotarget.27943

**Published:** 2021-04-13

**Authors:** Aditya Kulkarni, Joseph Ryan McDermott, Umesh Kathad, Rama Modali, Jean-Philippe Richard, Panna Sharma, Kishor Bhatia

**Affiliations:** ^1^Lantern Pharma, Inc., Dallas, TX 75201, USA; ^2^REPROCELL USA Inc., Beltsville, MD 20705, USA

**Keywords:** non-small cell lung cancer, acylfulvene, alkylating agent, PTGR1, LP-184

## Abstract

More than 40% of non-small cell lung cancer (NSCLC) patients lack actionable targets and require non-targeted chemotherapeutics. Many become refractory to drugs due to underlying resistance-associated mutations. KEAP1 mutant NSCLCs further activate NRF2 and upregulate its client PTGR1. LP-184, a novel alkylating agent belonging to the acylfulvene class is a prodrug dependent upon PTGR1. We hypothesized that NSCLC with KEAP1 mutations would continue to remain sensitive to LP-184. LP-184 demonstrated highly potent anticancer activity both in primary NSCLC cell lines and in those originating from brain metastases of primary lung cancers. LP-184 activity correlated with PTGR1 transcript levels but was independent of mutations in key oncogenes (KRAS and KEAP1) and tumor suppressors (TP53 and STK11). LP-184 was orders of magnitude more potent *in vitro* than cisplatin and pemetrexed. Correlative analyses of sensitivity with cell line gene expression patterns indicated that alterations in NRF2, MET, EGFR and BRAF consistently modulated LP-184 sensitivity. These correlations were then extended to TCGA analysis of 517 lung adenocarcinoma patients, out of which 35% showed elevated PTGR1, and 40% of those further displayed statistically significant co-occurrence of KEAP1 mutations. The gene correlates of LP-184 sensitivity allow additional personalization of therapeutic options for future treatment of NSCLC.

## INTRODUCTION

KEAP1, KRAS, TP53 and STK11/LKB1 are among the commonly altered genes with considerable clinical prevalence in non-small cell lung cancers (NSCLC). Alterations such as amplification, loss, missense mutations, splice site mutations or deleterious truncations in these genes account for greater than 40% of NSCLC cases [1, 2]. KRAS and KEAP1 promote NSCLC pathogenesis [3, 4] owing to their constitutive activation as oncogenes whereas TP53 and STK11 contribute to tumorigenesis upon inactivation of their tumor suppressor function [5, 6]. There is little overlap or redundancy in the interaction networks and signaling pathways in which these gene products operate, making it difficult to develop any single class of drugs retaining efficacy in these molecularly segregated NSCLC subtypes.

There is a high unmet need for effective therapies for NSCLC harboring mutations in these genes that have not only been considered undruggable till date but also are associated with loss of efficacy or resistance to multiple therapeutic strategies, at least in frontline regimens. For such NSCLC patients there is no specific approved targeted therapy option or standard chemotherapy, and alternative therapies are weakly to moderately effective. Patients with such tumors currently depend on chemotherapy agents including DNA damaging or alkylating agents. However, resistance to such agents including platinum compounds, taxanes and antimetabolites also develops. Newer drugs that will be more potent and remain efficacious in NSCLC with such mutations could lead to better alternate or combinatorial therapies.

Here, we describe the characteristics of LP-184 (Supplementary Figure 1), a next generation member of the acylfulvene class of prodrugs in the context of its anti-tumor activity in NSCLC cell line models [7–9]. LP-184 is currently in preclinical development focused on selected solid tumor indications including NSCLC. The overall goal of this work was to determine a range of NSCLC settings that LP-184 might be optimally positioned in. We profiled primary and metastatic *in vitro* models of NSCLC for their sensitivity to LP-184 as well as standard of care agents, evaluated gene correlates of LP-184 response, and obtained evidence on *in vivo* anti-tumor effect of LP-184.

Acylfulvenes have been derived from cytotoxic agents called Illudins, isolated from Jack-o-Lantern mushroom (*Omphalotus illudens*), that retain and improve the cytotoxicity of parent Illudins for use as anticancer agents. Mechanisms of acylfulvene cytotoxicity include nucleotide/amino acid specific-alkylation of DNA/RNA or protein, resulting in cell-cycle arrest and apoptosis, generation of reactive oxygen species and the chemical modification of various intracellular proteins, as well as inhibition of cytosolic redox-regulating thiol-containing proteins such as glutathione reductase, thioredoxin reductase, and thioredoxin [10–14]. Acylfulvene anti-tumor activity appears to be based on activation through reductive mechanisms that are mediated by enzymes such as Prostaglandin Reductase 1 (PTGR1) [15]. Activated acylfulvenes can oxidize various cellular thiols, as well as create DNA adducts that disrupt DNA and RNA synthesis. Resolution of these adducts is reported to exclusively proceed via transcription-coupled nucleotide excision repair (TC-NER) [16, 17].

Further, PTGR1 is known to be upregulated in tumors with deregulated NRF2, including in tumors with mutations in KEAP1 [18, 19]. Mutated KEAP1 and concomitant decreased KEAP1 activity in cancer cells induces greater nuclear accumulation of NRF2, causing enhanced transcriptional induction of antioxidants, xenobiotic metabolism enzymes, and drug efflux pumps, thereby rendering KEAP1 mutations predictive of chemotherapy resistance in NSCLC patients. The KEAP1-NRF2-PTGR1 axis is thus a critical determinant of therapy outcomes, and corresponding pathway aberrations provide an explanation for poor clinical outcomes observed in NSCLC [20, 21]. Similarly, therapies that counteract the oncogenic effects of mutant KRAS are still in development, as lung cancers driven by mutant KRAS remain among the most refractory to available treatments [22]. The identification of a trend toward detrimental overall survival among a subset of platinum-treated NSCLC patients harboring co-occurring KRAS and STK11 mutations could label a more aggressive molecular subtype of NSCLC [23]. NSCLC with TP53 alterations has been reported to carry a worse prognosis and may be relatively more resistant to chemotherapy and radiation [5]. We therefore investigated LP-184 sensitivity in NSCLC cell lines harboring individual or concomitant mutations in KEAP1, KRAS, TP53 and STK11.

We sought to assess LP-184 activity in a panel of selected NSCLC adenocarcinoma cell lines, determine associations between genomic and transcriptomic profiles and responses of cell lines tested, and compare *in vitro* potency of LP-184 with that of approved chemotherapy agents. We performed pathway enrichment and transcription factor regulation of genes differentially expressed across subsets of sensitive and insensitive NSCLC cell lines. We further investigated LP-184 response in a xenograft tumor model of lung cancer. We also identify and quantify molecular brackets corresponding to predicted LP-184 responders from clinical data analyses.

## RESULTS

**Figure 1:**
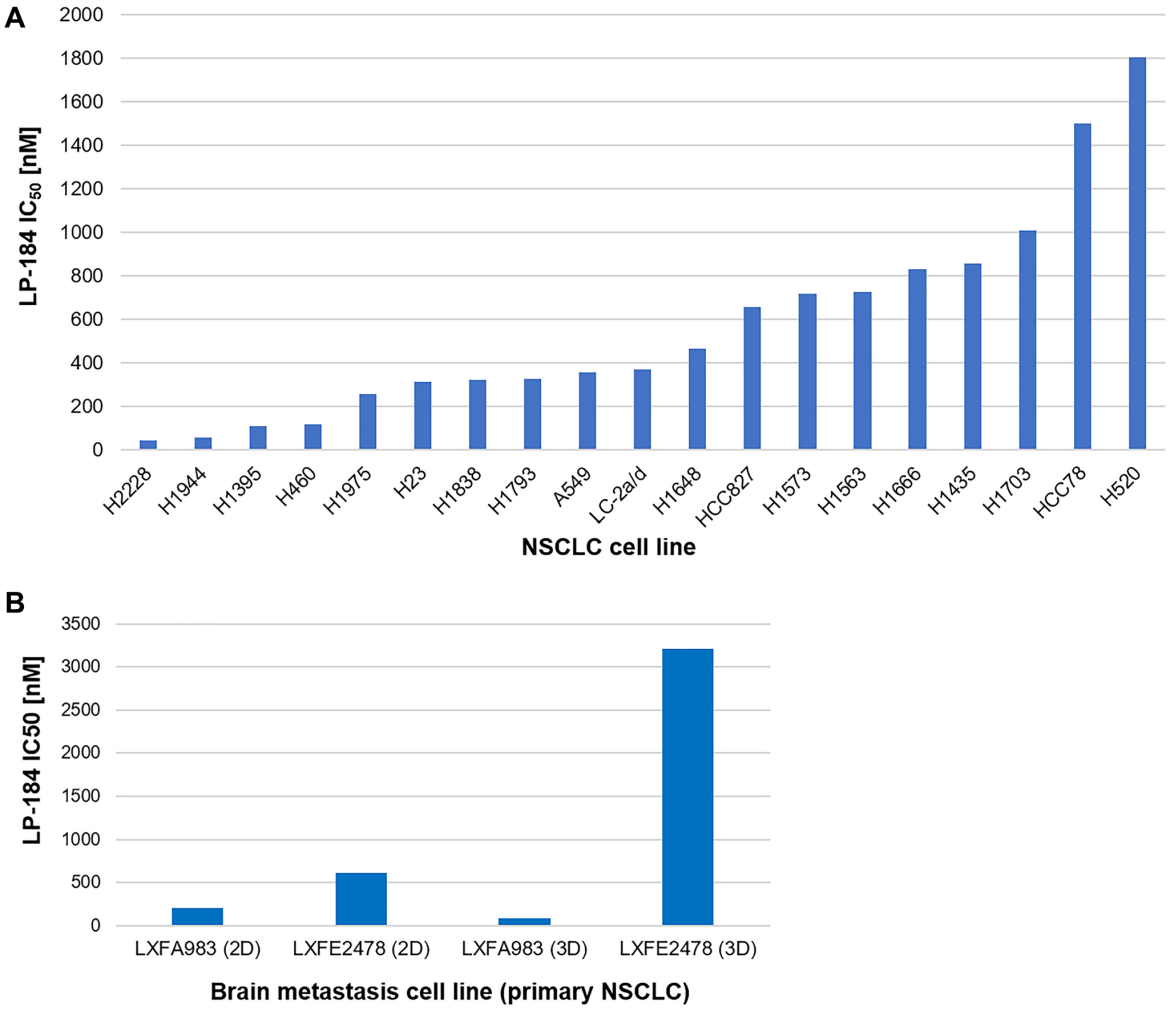
LP-184 shows broad anti-tumor cytotoxicity across a panel of NSCLC derived cell lines. (**A**) This bar graph demonstrates the range of NSCLC cell line sensitivity to LP-184, technically performed in triplicate wells (represented in Supplementary Figures 2–10), plotted in terms of nanomolar IC_50_ on Y axis and respective cell line IDs on X axis. (**B**) This bar graph demonstrates the sensitivity range of brain metastasis cell lines originating from primary lung cancers to LP-184, technically performed in duplicate wells (represented in Supplementary Figures 11–14), plotted in terms of nanomolar IC_50_ on Y axis and respective cell line IDs on X axis.

19 human NSCLC cell lines representing diverse molecular, demographic, and histological features (Supplementary Tables 1, 2) were treated with LP-184 in a 96 well format in triplicate wells, across concentrations ranging from 14 nM to 10 μM. Compound treatment was carried out for 72 hours and cell viability was assayed using Promega’s CellTiter Fluor reagent. Drug sensitivity was measured in terms of IC50 value generated from the dose response curve plotted in GraphPad Prism, as shown in Figure 1A. Representative dose response curves are depicted in Supplementary Figures 2–10 and IC50 values listed in Supplementary Table 3A. Overall, LP-184 exhibited strong nanomolar potency in the majority of NSCLC cell lines tested, indicating broad anti-tumor cytotoxicity in this panel. In these 19 NSCLC cell lines, the IC50 range was 45 to 1805 nM, with median IC50 of 371 nM and mean IC50 of 571 nM.

We extended this study of LP-184 responses in primary NSCLC cell lines to *in vitro* models of brain metastases originating from primary lung cancers. Two such models LXFA 983 and LXFE 2478 were tested for their sensitivity to LP-184 both in 2D and 3D culture systems. For 2D cultures, the CellTiter-Glo^®^ assay provided a cell viability readout whereas for 3D cultures, the 3D Clonogenic assay provided a vital stain-based colony formation readout. As shown in Figure 1B, LP-184 retained efficacy in these models, ranging between IC50 of 88 nM and 3209 nM. Representative dose response curves are depicted in Supplementary Figures 11–14 and IC50 values listed in Supplementary Table 3B. The translational relevance of these results is underscored by the blood brain barrier crossing property of LP-184. An *in silico* analysis of predicted ADMET properties of LP-184, using the AdmetSAR 2.0 web tool [24], yielded a high blood brain barrier permeability probability score of 0.9694, comparable to the published score of 0.9879 for Temozolomide accessed from the DrugBank database [25]. Temozolomide is one of the classic standard of care agents approved for multiple brain tumor types.

**Figure 2:**
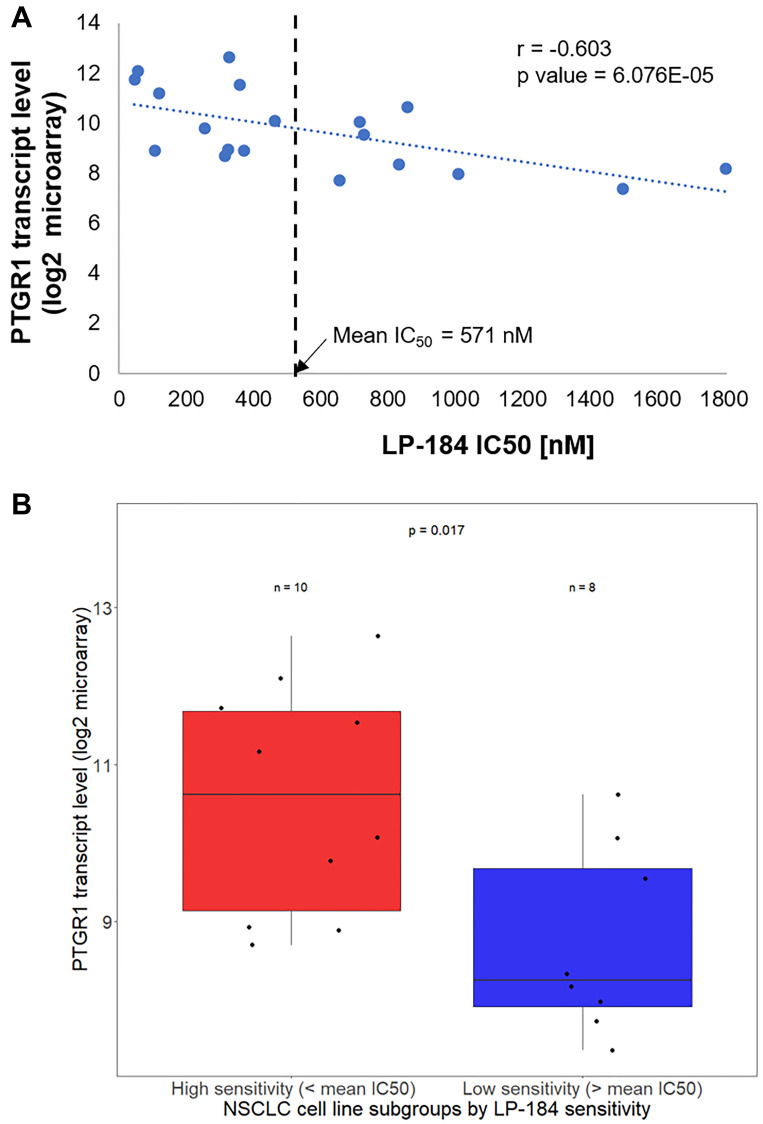
LP-184 sensitivity in NSCLC cell lines correlates with PTGR1 transcript levels. (**A**) This scatter plot shows the correlative trend between LP-184 sensitivity on X axis and PTGR1 transcript levels across the 19 NSCLC cell lines tested. (**B**) This box plot statistically compares PTGR1 transcript levels between LP-184 high and low sensitive cell lines.

As a next generation member of the class of acylfulvene prodrugs, the anti-tumor activity of LP-184 is likely to be dependent upon the oxidoreductase activity of PTGR1. Consistent with this background, as depicted in Figure 2A, we found that LP-184 sensitivity in the NSCLC cell lines tested correlates with PTGR1 transcript levels (Pearson Correlation Coefficient, *r* = –0.603, *p value* 6.076E-05). We used the mean LP-184 IC50 value of 571 nM to divide the NSCLC cell lines in high and low sensitivity groups. PTGR1 expression was compared among two groups of NSCLC cell lines tested: 11 cell lines with LP-184 IC50 < 571 nM and 8 cell lines with IC50 > 571 nM. As displayed in Figure 2B, PTGR1 transcript levels turned out to be significantly different (*p* value 0.017) in these cell line groups.

To investigate the influence of KEAP1 mutations on PTGR1 transcript levels in the panel of 19 NSCLC cell lines tested, PTGR1 expression was compared, among two groups of cell lines: 7 cell lines with KEAP1 mutation and 12 cell lines without KEAP1 mutation. We found that the difference in PTGR1 expression between KEAP1 mutant and wild type cell lines is significant upon one-tailed *t-test* analysis (*p value* 0.0253) and just falls short of achieving statistical significance upon two-tailed *t-test* analysis (*p* value 0.0506). In comparison, PTGR1 expression is entirely independent of mutations in KRAS, TP53 and STK11 which are commonly altered in NSCLC but currently lack effective targeted therapy options (Supplementary Figure 15).

To interrogate the impact of mutations, in key oncogenes KRAS and KEAP1 and tumor suppressors TP53 and STK11 that underly a large fraction of undruggable NSCLC, on LP-184 sensitivity, we compared mean LP-184 IC50 between subsets of wild type and mutant cell lines for the individual genes. As plotted in Supplementary Figure 16, we observed that LP-184 sensitivity is independent of mutations in these genes, suggesting that LP-184 retains its activity even in the presence of deleterious mutations in these genes that would otherwise be associated with chemotherapy resistance in NSCLC.

**Figure 3:**
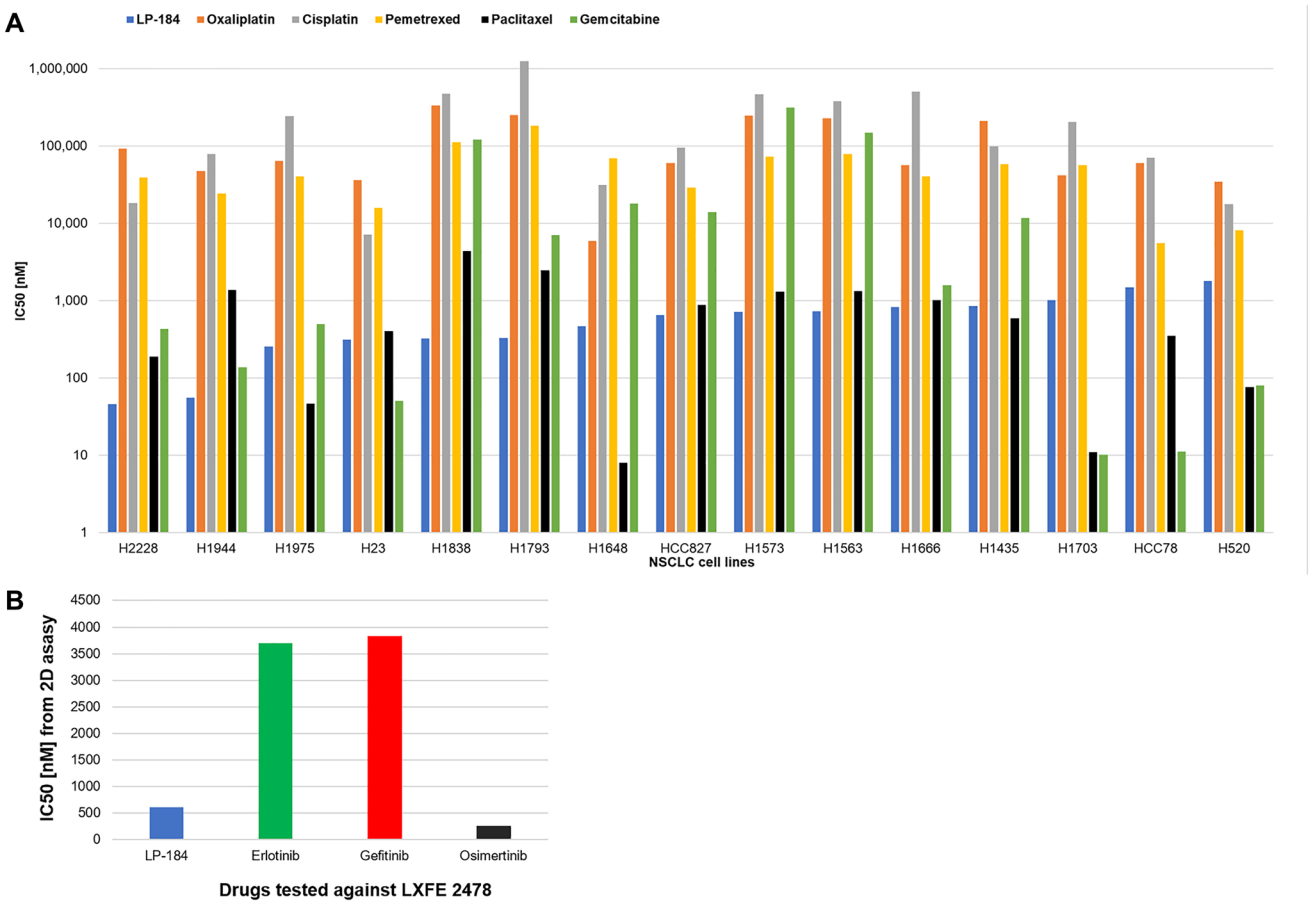
LP-184 is generally more potent *in vitro* than commonly used chemotherapy agents. (**A**) In 15 selected NSCLC cell lines (X axis), potency in terms of nanomolar IC50s on log scale (Y axis) is compared among LP-184 obtained in our study, and Oxaliplatin, Cisplatin, Pemetrexed, Paclitaxel and Gemcitabine obtained from the GDSC database. (**B**) In the brain metastasis model LXFE 2478, originating from primary lung cancer, 2D *in vitro* activity in terms of nanomolar IC50s (Y axis) is compared among LP-184 obtained in our study, and Erlotinib, Gefitinib and Osimertinib cited in [28].

We further performed a broad comparison in selected NSCLC cell lines, of our LP-184 responses with previously published responses of commonly prescribed standard chemotherapeutics obtained from the GDSC database. Among those were Oxaliplatin and Cisplatin that also act as DNA alkylating agents but likely via a mechanism not overlapping with that perceived for LP-184 [26, 27]. We also obtained known responses to the antimetabolites Pemetrexed and Gemcitabine, also currently considered as a standard therapy option for NSCLC. Lung adenocarcinomas can also be treated with taxanes. Publicly available IC50 data on Oxaliplatin, Cisplatin, Pemetrexed, Paclitaxel and Gemcitabine gathered after a 72-hour treatment as with LP-184, and typically reported as mean values without standard error were obtained from the GDSC database. In this analysis of relative cytotoxicity across selected NSCLC cell lines, as shown in Figure 3A, LP-184 turned out to be up to 3800 times more potent than some of these chemotherapeutics approved for medical use in NSCLC (Supplementary Table 4). The brain metastasis model LXFE 2478 harbors a heterozygous EGFR-activating mutation, i.e., EGFR exon 20 insertion (M766_A767insASV). The patient from whom this model was derived was reported to be resistant to radiotherapy, cisplatin/erlotinib/pemetrexed combination and PD-L1 antibody treatments. We compared the published *in vitro* 2D efficacy of various EGFR inhibitors [28] in this model with that of LP-184 under similar conditions, and found that LP-184 is about 6 times more potent than earlier generation EGFR inhibitors Erlotinib and Gefitinib whereas about 2.4 times less potent than the latest generation EGFR inhibitor Osimertinib, thereby placing LP-184 within this spectrum (Figure 3B).

**Figure 4:**
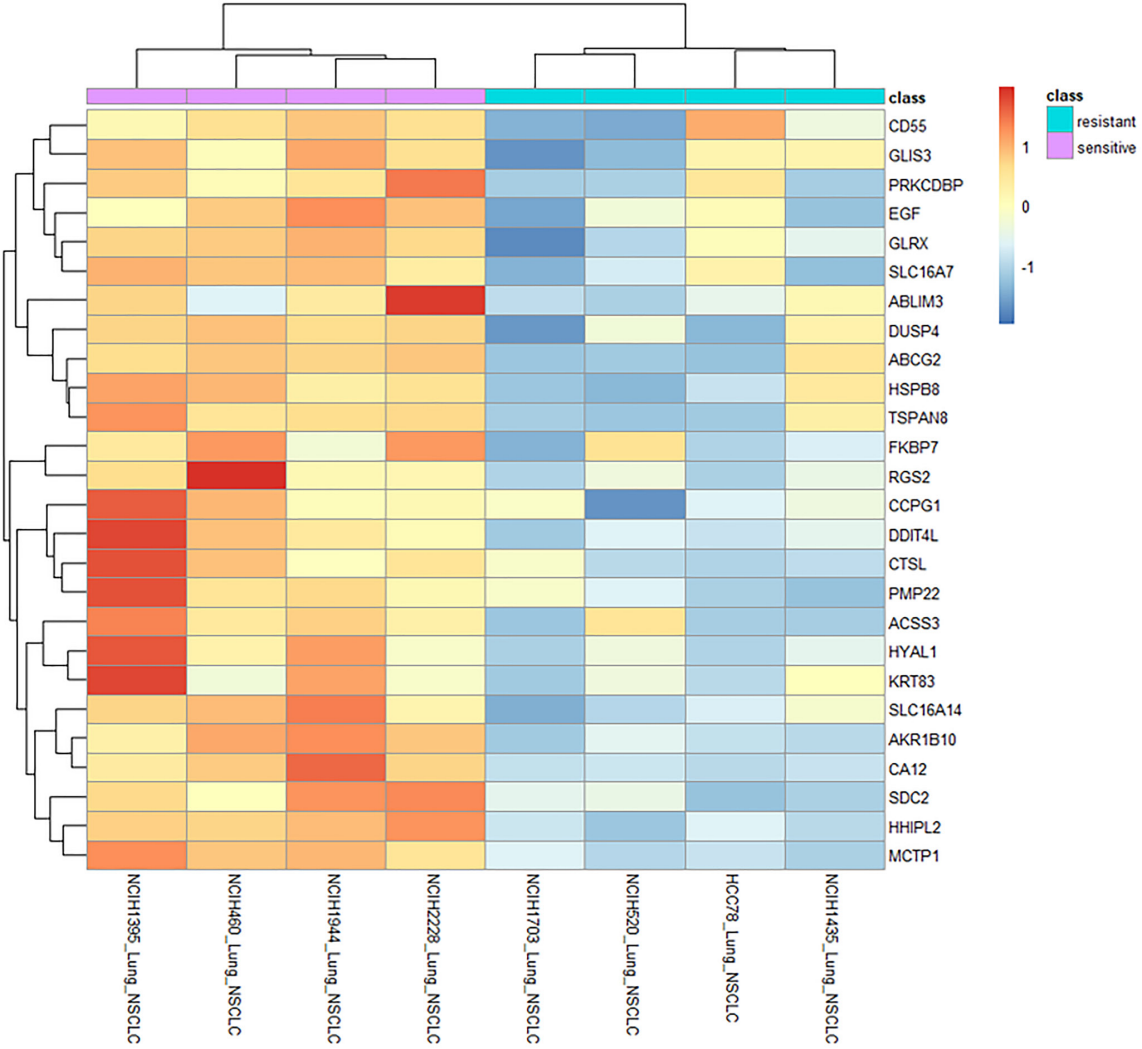
Gene correlation and expression pattern analyses across differentially sensitive NSCLC cell lines highlights key processes potentially linked with LP-184 activity. Heatmap shows expression patterns of genes (vertical axis) correlated significantly with LP-184 sensitivity (*p* value < 0.01) and differing by >2 log2 units when comparing the 4 most sensitive (pink bar) and 4 most resistant (cyan bar) NSCLC cell lines (horizontal axis).

To identify which genes were most strongly associated with LP-184 sensitivity, we examined the most significant (*p* < 0.01) of the genes correlated to sensitivity in the top 4 NSCLC cell lines at the extremes of sensitivity or insensitivity. Genes that were more expressed in the sensitive lines (with mean log2 expression values greater than 2 times the mean values of the resistant lines) are shown in a clustered heatmap in Figure 4. These 26 genes may be markers and/or causal factors of LP-184 sensitivity, at least in the NSCLC cell line panel evaluated. Genes with greatest differential expression between the extreme sensitive and resistant NSCLC cell lines can potentially reveal new insights into LP-184 mechanism of action in NSCLC. Of particular interest in this list are AKR1B10 and EGF. Aldo-Keto Reductase family member B10 (AKR1B10) had a more consistent association of higher expression in sensitive cell lines. EGF is the ligand of EGFR, which further supports the relationship between the EGFR signaling pathway and LP-184 response.

We next investigated gene-expression pattern comparisons across LP-184 sensitive and resistant NSCLC cell lines. The assayed NSCLC cell line transcriptomes were correlated to the z-score of LP-184 sensitivity such that higher z-score values correspond to increased sensitivity. There were 115 positively correlated transcripts and 90 negatively correlated transcripts having an absolute Pearson’s Correlation Coefficient (PCC) over 0.5. As elucidated in Supplementary Figure 17, we separately analyzed pathway enrichment of both groups in the Wikipathways database. Positively-associated transcripts were overrepresented in pathways related to NAD+ production and nuclear receptor pathways. Negative associations were enriched in pathways of the SMARCB1 tumor suppressor, DNA methylation, and the proteasome.

Analysis of regulatory factors that may be responsible for the expression profiles of the positive and negative gene groups was performed by determining their overlap with annotated gene-set libraries based on perturbations in either kinase signaling (Supplementary Figure 18) or transcription factors (Supplementary Figure 19). Kinase perturbations were examined by the enrichment of the gene groups in sets that contain 300 differentially expressed genes for different kinase alterations, including gain and loss of function experiments. Similarly, genes differentially expressed after transcription factor perturbations were used to test for enrichment among the positively correlated expression group. Supporting the validity of this approach, the analysis confirmed that NRF2 is required for PTGR1 expression, as NRF2 loss identified PTGR1 (among other genes) in the NRF2-knockdown-downregulated gene set, as expected. Several kinase cascades showed a consistent pattern of regulation, where enrichment in gain or loss of function experiments showed consistent changes in the direction of their associations. For example, in the group negatively associated with LP-184 sensitivity, gene sets of downregulated transcripts after MET knockout were the most significantly enriched, whereas in the positive-association group, the gene set of upregulated transcripts after MET knockout was the most enriched. Additionally, gene sets from separate MET knockouts produced consistent results. Members of the PI3K/AKT pathway also showed similar consistency.

**Figure 5:**
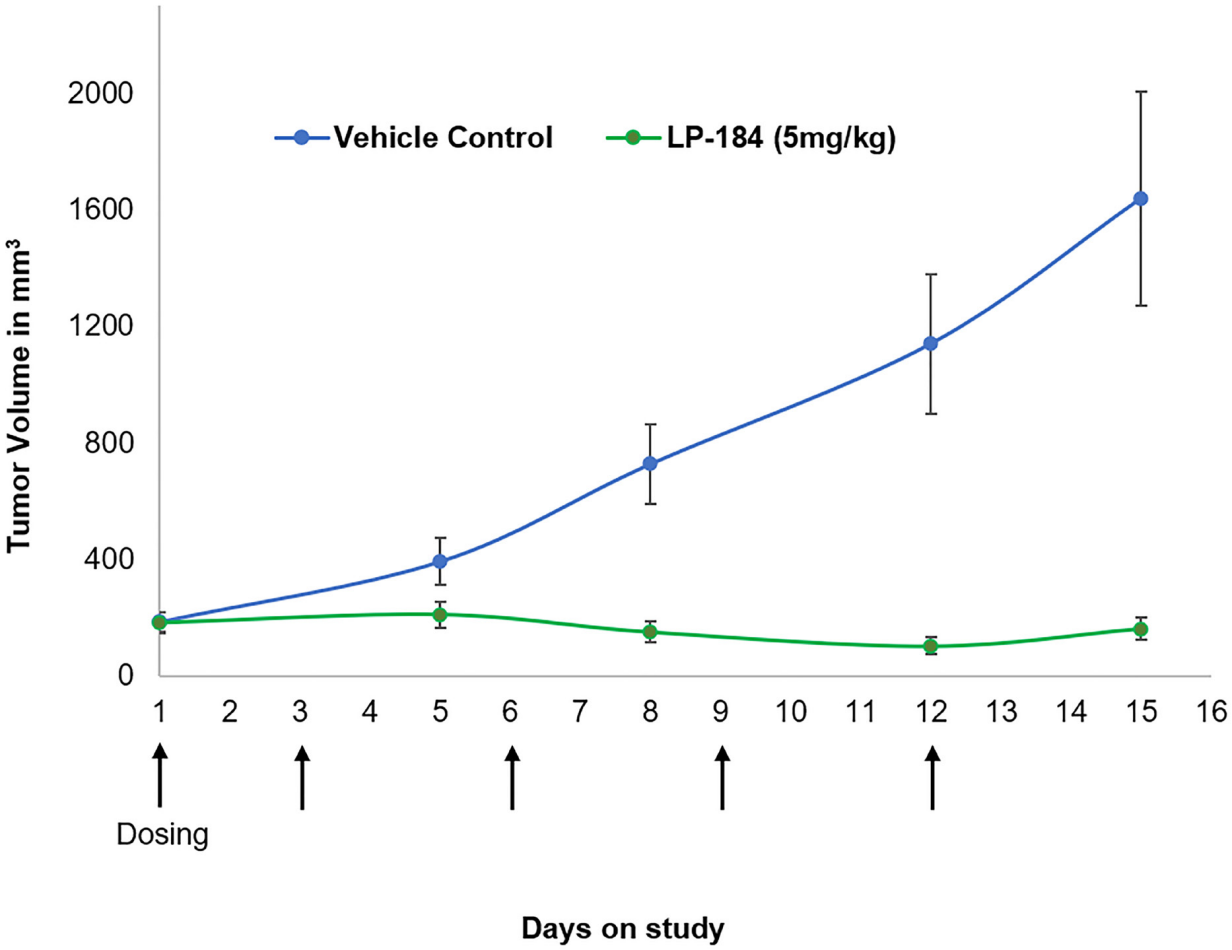
Activity of LP-184 in the H460 nude mouse xenograft model. Y axis shows tumor volume in mm^3^. LP-184 or vehicle control was administered intraperitoneally on the days indicated by the arrows on the X axis (*N* = 10 in each group).

LP-184 anti-tumor response was evaluated *in vivo* in the NCI-H460 lung tumor model as a subcutaneous xenograft in nude mice. Ten mice were included in the vehicle control and treatment groups. As shown in Figure 5, LP-184 treatment was conducted using a regimen of five 5 mg/kg injections administered intraperitoneally on days 1, 3, 6, 9 and 12. The individual mouse tumor volumes and body weights on the sampling days are listed in Supplementary Tables 6 and 7. This treatment yielded statistically significant differences (Supplementary Table 8) in mean tumor volumes of vehicle control and treatment groups on days 8, 12 and 15. LP-184 thus demonstrated anti-tumor efficacy in a lung cancer model, supporting continued investigations across various NSCLC subtypes and regimens.

**Figure 6:**
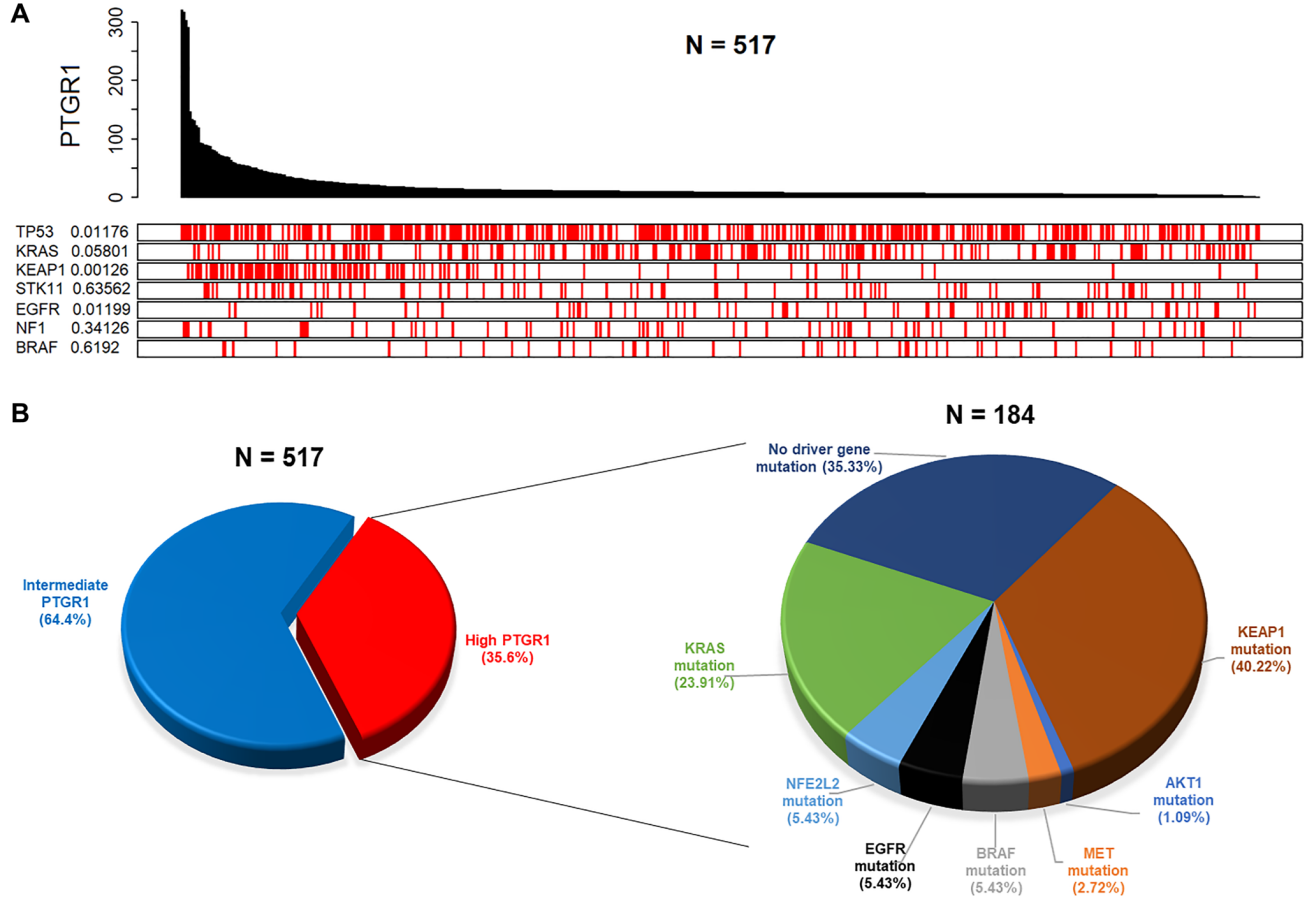
Clinical data analyses from TCGA reveal distinct patient subgroups with elevated PTGR1 that are likely to be predicted responders to LP-184. (**A**) Analysis of clinical data on 517 lung adenocarcinoma patients shows frequency of mutated genes across the observed PTGR1 relative gene expression range. The value adjacent to the highly mutated gene is a two-sided permutation test *p*-value of PTGR1 relative gene expression between driver mutated (red) and non-mutated (white) samples. (**B**) Analysis of clinical data on the same 517 lung adenocarcinoma patients as in (A) displays patient subsets by PTGR1 expression status, and mutational frequencies of selected driver genes within the PTGR1 elevated subset.

We further investigated PTGR1 expression status in relation to multiple relevant genes’ mutation status in clinical datasets of NSCLC. In an analysis of 533 NSCLC adenocarcinoma patient records from the TCGA portal, PTGR1 was found to be highly expressed in KEAP1 mutated samples. In the plot in Figure 6A, the value adjacent to the highly mutated gene is the permutation test *p*-value of PTGR1 relative gene expression between driver mutated (red) and not-mutated (white) samples. This result is the most statistically significant for KEAP1 (*p* value 0.00126), with PTGR1 being highly expressed in KEAP1 mutated samples. Similarly, in a complementary analysis exploring clinical data from TCGA, we determined that greater than 35% of a total of 517 NSCLC adenocarcinoma patients expressed elevated PTGR1 transcript levels (Figure 6B). Within this PTGR1 high subset, there were distinct brackets enriched in KEAP1, KRAS, BRAF, EGFR, NRF2, MET, and AKT1 mutations. The pie chart on the right represents the percentages of PTGR1 high patients, filtered for presence of damaging mutations in the individual selected driver genes, that are not necessarily mutually exclusive. Populations harboring such mutations co-occurring with elevated PTGR1 levels potentially represent molecularly defined NSCLC patient subgroups likely to benefit from an LP-184 based regimen.

## DISCUSSION

We are advancing LP-184, a small molecule anticancer drug candidate using a precision medicine approach, by defining the responsiveness of a tumor to LP-184 based on its gene expression and mutation pattern. LP-184 is a novel small molecule belonging to the acylfulvene drug class [9] related to Illudin S, a toxin that occurs naturally in certain mushrooms [29]. Chemically, LP-184 is N-hydroxy-N-(methylacylfulvene)urea.

In preliminary studies, LP-184 efficacy had been established in the NCI60 cell line panel, with NSCLC emerging as the most prominent sensitive cancer type as 4 of the top 10 sensitive cell lines were NSCLC cell lines (unpublished data). LP-184 showed clear differential sensitivity across two broad cancer lineages: solid tumor cell lines were on average more responsive, relative to cancer cell lines of hematological origin which were particularly resistant to LP-184, consistent with the expression pattern of PTGR1 in cancers of these lineages. We have demonstrated that LP-184 has nanomolar potency with IC50s < 500 nM in 11 of the 19 NSCLC cell lines tested. This indicates broad anti-tumor activity in NSCLC.

Even across 19 cell lines representing one cancer subtype - NSCLC adenocarcinoma - where the spectrum of LP-184 sensitivity (~40 fold between the most and least sensitive NSCLC cell line, Figure 1A) is not ranging as widely as that observed across multiple cancer types and lineages (~700 fold between the most and least sensitive solid tumor cell line, obtained from unpublished work), PTGR1 gene expression is still moderately but significantly correlated with LP-184 sensitivity (Figure 2A). This suggests that PTGR1 is likely to be a primary causal determinant underlying LP-184 sensitivity prediction. This is also reflected in the observation that using a threshold of 571 nM as the mean IC50, the two high and low sensitive cell line subgroups can be statistically stratified (*p* value 0.017) by differential PTGR1 transcript levels (Figure 2B).

20–40% of adults with non-small cell lung cancer go on to develop brain metastases at some point [30]. Besides surgery and stereotactic radiation, newer treatments, such as immunotherapies and targeted therapies that can cross the blood-brain barrier, may be recommended as complementary treatment options. LP-184 appears to be a promising candidate in this armamentarium of drugs based on its efficacy in brain metastasis models of lung cancer and potential to penetrate the blood-brain barrier. Its observed activity in an EGFR-activated brain metastasis tumor model (Figure 1B) is also corroborated by outputs from bioinformatics analyses (Supplementary Figure 18).

Further, LP-184 sensitivity appeared to remain unaffected by presence of loss of function mutations in key oncogenes KEAP1 and KRAS and tumor suppressors TP53 and STK11 (Supplementary Figure 16). Evaluating two cell lines identified as harboring concomitant mutations in all these four genes (H23 and H1573) along with two comparator cell lines identified as “wild type” for these four genes (H1703 and H1975), we did not find any significant difference in LP-184 sensitivity between these cell line subgroups, although this is a small sample size.

*In vitro* potency of LP-184 turns out to be minimally affected, if at all, by presence of mutations in key oncogenes/tumor suppressors (Supplementary Figure 16) that would otherwise be related to resistance to other known drugs or considered undruggable due to unsuccessful attempts at direct targeting. The KEAP1-NRF2-PTGR1 interaction network and implications of its deregulation in NSCLC has not been sufficiently recognized and clinically targeted till date. KRAS positive NSCLC patients have recently been considered to be eligible for immune checkpoint blockade plus chemotherapy as first-line regimen as a way to activate anti-tumor immunity but clinical outcomes have been varying [31]. Coexisting alterations in KEAP1 and STK11 among other genes define a subset of lung adenocarcinoma unresponsive to immunotherapy [32]. LKB1/STK11 mutations in association or not with KRAS were also reported to be related to a lack of response to immunotherapy [33]. TP53 mutation in NSCLC is also associated with poor response to targeted therapy [5]. These may represent either overlapping or discrete molecular subsets that are likely to benefit from LP-184 based therapy.

LP-184 exhibits orders of magnitude greater *in vitro* efficacy than standard of care agents used in NSCLC (Figure 3A, Supplementary Table 4). Similarly, its *in vitro* activity is comparable to that of approved EGFR inhibitors tested in a brain metastasis model. This sensitivity differential may also indicate improved tolerability and tumor selectivity of LP-184 relative to existing drugs in multiple settings. Cytotoxicity data on non-target comparator cell lines from previously published as well as unpublished work suggests that LP-184 has a broad therapeutic window relative to hematological cells (Supplementary Table 5).

We characterized gene expression associations in NSCLC with sensitivity to LP-184 to determine if any consistent gene expression pattern exists, and if these associations are functionally related to drug sensitivity (Supplementary Figures 17–19). Pathways enriched by the most positively correlated genes to LP-184 sensitivity most correlated genes to LP-184 sensitivity uncovered various pathways, some of which relate to LP-184 mechanisms and cancer growth. Pathways related to NAD+ were enriched due to the positively correlated genes of NAMPT and TDO2. Acylfulvene prodrugs such as LP-184 are NADPH dependent [11, 13], and the balance of NADPH is also affected by enzymes that influence NAD/NADH status. NAMPT is the major NAD+ biosynthetic enzyme and has been implicated in cancer stemness, as well as redox homeostasis and DNA repair [34]. TDO2 is expressed predominantly in the central nervous system in normal tissue but is overexpressed in various cancers and linked to promotion of survival and immune resistance. TDO2 is connected to NAD+ generation through tryptophan catabolism in the first step of the kynurenine pathway [35]. Enrichment of NAD+ pathways suggests the possibility of indirect processes of NAD metabolism influencing LP-184 pro-drug activation, or modifying sensitivity through changes in cellular redox balance.

With negatively associated genes, there was a repeated pattern of epigenetic regulatory pathways enriched in the Wikipathways library. Interestingly, the most enriched pathway in this group was the SMARCB1 tumor suppressor pathway, suggesting this pathway leads to gene expression that is unfavorable to LP-184 response. SMARCB1 is a SWI/SNF chromatin regulatory tumor suppressor, and mutations in it or similarly functioning members of this family are relatively common in many cancer types, including lung [36]. DNA-methylation-related pathways were also prominent in the enrichment results of negatively-correlated genes, owing to a strong anti-correlation of TET1 and TET2 transcripts to LP-184 response. TET1 and TET2 function is necessary for normal maintenance of the epigenome, and TET1 deficiency has been recently reported to promote resistance to DNA damage [37]. Whether and how these pathways relate to LP-184 anti-tumor cytotoxicity, or specifically relate to the PTGR1-mediated activation of LP-184, warrants further investigation.

NRF2 is known to upregulate PTGR1, which was confirmed by enrichment analysis of transcriptional regulators of genes positively correlated with LP-184 response. As expected, PTGR1 was one of the most strongly positively associated genes to LP-184 sensitivity, and enrichment analysis of positively associated genes in a library of transcriptional regulators revealed that NRF2 loss significantly decreases PTGR1 expression, among others that are associated with drug sensitivity. Accurate identification of this validated association lends confidence that other significantly enriched transcription factors are true regulators of gene expression involved in LP-184 sensitivity. This also revealed that a significant set of LP-184 associated genes are MYC-dependent, which is of interest given the role of MYC in general- and NSCLC-tumor progression [1].

A similar enrichment analysis was performed on kinase cascade perturbation libraries. Sets of genes differentially expressed after various kinase perturbations were analyzed for the positive and negative gene groups. This repeatedly identified hits with various components in MET, AKT, and MAPK pathways. A symmetry of the influence of these pathways was revealed between different groups and the separate upregulated and downregulated libraries. MET knockdown and knockout upregulated genes associated with LP-184 sensitivity, suggesting MET activity suppresses their expression, while MET knockout downregulated expression of LP-184 anti-correlated genes, suggesting that anti-correlated gene expression is dependent on MET activity. These pathways are involved in progression of NSCLC and are reported to mediate acquired resistance to EGFR TKI therapy. We observed drug activation of EGFR was enriched with negatively associated genes to LP-184 sensitivity, suggesting EGFR activity suppresses genes that inhibit, or are negatively correlated to, LP-184 response. The association of this pathway is consistent with our finding that EGFR mutations were among the most frequent in NSCLC, given that most EGFR mutations are gain-of-function. When determining the genes most significantly differentially expressed between the extremely sensitive versus extremely resistant lines, we identified 26 genes with significant and substantially higher expression in sensitive lines, and EGF and AKR1B10 were in this group (Figure 4). These results suggest LP-184 is likely to be a promising candidate for treating TKI-resistant NSCLC. Similarly, AKR1B10, the other differentially expressed gene has been reported to be overexpressed 3.9-fold compared to normal tissue, leading it to be proposed as a prognostic marker [38]. NF-κB and Erk1/2 phosphorylation were dependent on AKR1B10 expression, and AKR1B10 was also required for invasion, adhesion, and proliferation *in vitro*. Potentially these traits play a causal role in the association of AKR1B10 with LP-184 sensitivity, however it is possible it plays a direct role in activation of the prodrug. Previously, AKR1B10 was proposed to be a biomarker for liver, lung, and other cancers, and shown to act as a carbonyl reductase on the chemotherapeutic daunorubicin [39]. AKR1B10 is in the NADP-dependent oxidoreductase domain superfamily, with PTGR1 [40]. AKR1B10 may thus have a similar role as PTGR1 in LP-184 prodrug activation. Future studies will test the direct and indirect hypothetical roles of AKR1B10 and EGFR signaling on LP-184 NSCLC response.

Thus far, determinants of response to any acylfulvene have not been explored outside of proteins directly involved in LP-184 activation and subsequent DNA damage repair. Our results may be leveraged to determine which cancer types or patients will most benefit from therapy. The finding that EGFR-signaling components, and other pathways related to therapeutic resistance and poor clinical outcomes, are associated with sensitivity indicates that predicted LP-184 responders may overlap with indications of therapeutic need.

LP-184 shows efficacy in terms of tumor regression in the *in vivo* xenograft tumor model H460 using the chosen regimen (Figure 5). The effective dose of 5 mg/kg used in this regimen did not cause acute or intolerable body weight loss under the conditions tested (Supplementary Tables 6 and 7). There is scope to expand the testing of additional models and dosing regimens to establish an optimized scheme for demonstrating safe, stable, and complete tumor regression with LP-184 treatment.

Gene changes in the positive-association group had a consistent relationship to BRAF perturbations. The set of transcripts down after BRAF knockdown were overrepresented in the positive-association group, and so were the transcripts up after BRAF overexpression. The genes which were enriched in up- and downregulated transcript sets for these BRAF perturbations were not redundant, as zero genes overlapped between both sets. This suggests BRAF activity may lead to expression of genes that increase LP-184 sensitivity, and this is further supported by our finding of BRAF being the 7th most mutated gene in a selected clinical dataset on NSCLC (Figure 6A). A similar connection is found with an EGFR-inhibitor downregulated gene set. The negative-association group was significantly enriched within the set of downregulated genes after EGFR activation. This likely indicates that EGFR activity inhibits expression of genes that are antagonistic to LP-184 cytotoxicity. Gain-of-function mutations in EGFR play a major role in lung cancer malignancy. As shown in Figure 6A, EGFR was the 5th most mutated gene in the NSCLC clinical dataset analyzed, and together suggests that this gain-of-function can enhance LP-184 sensitivity.

Our key findings demonstrate that the alkylating agent LP-184 has nanomolar potency in several NSCLC cell lines and is more potent than selected approved alkylating chemotherapeutics. Additionally, LP-184 has the potential to target tumors with elevated PTGR1 regardless of presence of other co-occurring mutations but is especially found to be effective in the background of clinically significant KEAP1 mutations. We propose further evaluation of LP-184 in multiple PTGR1 high NSCLC settings that may not necessarily be mutually exclusive, including in highly prevalent KEAP1 and KRAS mutant tumors (Figure 6), and in patients with lack of actionable targets or resistance-related genes with no effective therapy options available.

## MATERIALS AND METHODS

### Reagents

The compound LP-184 (molecular weight 304.34) is Lantern Pharma’s drug candidate. A working stock concentration of 10 mM in DMSO was used for all studies.

19 NSCLC cell lines listed in Supplementary Table 1 were obtained from the American Type Culture Collection (ATCC, Rockville, USA) at REPROCELL USA in August 2019 and cell line-based experiments completed in November 2019. Fewer than six months passed between resuscitation of cell line stock from the ATCC cell bank and final passage for experiments. ATCC uses morphology, karyotyping, and PCR based approaches to confirm the identity of human cell lines and to rule out both intra- and interspecies contamination. These include an assay to detect species specific variants of the cytochrome C oxidase I gene (COI analysis) to rule out inter-species contamination and short tandem repeat (STR) profiling to distinguish between individual human cell lines and rule out intra-species contamination.

2 models established from the brain metastasis originating from primary lung cancers, LXFA 983 (adenocarcinoma) and LXFE 2478 (adenosquamous) were tested in-house at Charles River Discovery Research Services, Germany. Tumor cells were grown at 37°C in a humidified atmosphere with 5% CO_2_ in RPMI 1640 medium, supplemented with 10% (v/v) fetal calf serum and 50 μg/ml gentamicin for up to 20 passages, and passaged once or twice weekly. Cells were harvested using TrypLE or PBS buffer containing 1 mM EDTA, and the percentage of viable cells was determined using a CASY Model TT cell counter (OMNI Life Science).

### CellTiter-Fluor^®^ cell viability assay

Each NSCLC cell line was thawed and after 24–48 hours in culture, the cells were harvested and counted. 5,000–10,000 cells were seeded in triplicate wells per test concentration with 100 μl of growth medium in each well of one tissue culture treated flat bottom black sided 96-well plate and incubated overnight in a cell culture incubator set to 37^o^C with 5% CO_2_. The day after, the medium was replaced with the drug added to final concentrations of 14 nM, 41 nM, 123 nM, 370 nM, 1.11 μM, 3.33 μM, and 10 μM, along with triplicate wells treated with the vehicle control DMSO in which LP-184 was solubilized. After 72 hours of drug treatment, cell viability was measured using Promega’s *CellTiter-Fluor*^®^ assay. 50 μl of GF-AFC reagent (10 μl of GF-AFC substrate diluted in 5 ml of assay buffer for each 96 well plate) was added to each well, mixed and incubated for 30 minutes at 37°C. Fluorescence signal reflecting live cell count was detected using 400 excitation/505 emission wavelength settings on a plate reader. Fluorescence signal from no cell control wells was subtracted as background from the signal of each well with cells. These baseline-adjusted relative fluorescence units were normalized to those of the DMSO treated wells being considered as 100% signal. Dose response curves and IC50s were generated using GraphPad Prism.

### CellTiter-Glo^®^ cell viability assay

LXFA983 and LXFE2478 cells as 2D models were harvested from exponential phase cultures, counted, and plated in 96 well flat-bottom microtiter plates at a cell density depending on the cell line’s growth rate (4,000–20,000 cells/well depending on the cell line’s growth rate, up to 60,000 for hematological cancer cell lines) in RPMI 1640 medium supplemented with 10% (v/v) fetal calf serum and 50 μg/ml gentamicin (140 μl/well). Cultures were incubated at 37°C and 5% CO_2_ in a humidified atmosphere. After 24 h, 10 μl of test compound LP-184 or control medium were added and left on the cells for another 72 h. LP-184 was serially diluted in DMSO, transferred in cell culture medium, and added to the assay plates by using a Tecan Freedom EVO 200 robotic platform. The DMSO concentration was kept constant at 0.3% v/v across the assay plate. Final concentrations of LP-184 were in the range of 1 nM – 10 μM. Every 96 well plate included six DMSO-treated control wells and drug-treated wells in duplicate at 9 concentrations. Luminescence signal from no cell control wells was subtracted as background from the signal of each well with cells. These baseline-adjusted relative luminescence units were normalized to those of the DMSO treated wells in six replicates being considered as 100% signal. Viability of cells was quantified by the CellTiter-Glo^®^ cell viability assay (Promega G8462). After incubation of cells, the CellTiter-Glo^®^ One Solution Assay reagent was brought to ambient temperature. Next, 100 μl of CellTiter-Glo^®^ One Solution Assay reagent were added to each well. Plates were shaken for 2 minutes to induce cell lysis and incubated for 20 minutes prior to reading luminescence (LU) by using the EnVision^®^ Xcite multilabel plate reader (Perkin Elmer). Sigmoidal concentration-response curves were fitted to the data points (test-versus-control, T/C values) obtained for each tumor model using 4 parameter non-linear curve fit (Charles River DRS Datawarehouse Software). IC50 values are reported as relative IC50 values, being the concentration of test compound that give a response halfway between the top and bottom plateau of the sigmoidal concentration-response curve (inflection point of the curve).

### 3D clonogenic assay

Patient-derived tumor xenografts LXFA983 and LXFE2478 were passaged as subcutaneous xenografts in NMRI nu/nu mice. At a tumor volume of 400–1000 mm^3^ tumor-bearing mice were sacrificed and tumors were collected under sterile conditions without delay according to relevant SOPs and animal welfare guidelines published by the FELASA and the GV-SOLAS. Tumors were mechanically disaggregated and subsequently incubated with an enzyme cocktail consisting of collagenase type IV (41 U/mL), DNase I (125 U/mL), hyaluronidase type III (100 U/mL), and dispase II (1 U/mL) in RPMI 1640 medium at 37°C for 60–120 minutes. Cells were passed through sieves of 100 μm and 40 μm mesh size and washed with RPMI 1640 medium. The percentage of viable cells was determined in a Neubauer-hemocytometer using trypan blue exclusion. Aliquots of the primary tumor cell suspension were frozen down and stored in liquid nitrogen vapor phase. The 3D clonogenic assay was carried out in 96 well plate format using ultra low attachment plates. For each test, a frozen aliquot of tumor cells prepared from tumor xenografts was thawed and assay plates were prepared as follows: each test well contained a layer of semi-solid medium with tumor cells (50 μl), and one layer of medium supernatant (100 μl), with or without test compound LP-184. Tumor cells were seeded in soft-agar medium, consisting of 50 μl/well Iscove’s Modified Dulbecco’s Medium (IMDM), supplemented with 20% (v/v) fetal calf serum, 50 μg/ml gentamicin, and 0.4% (w/v) agar. Cultures were incubated at 37°C and 7.5% CO_2_ in a humidified atmosphere. After 24 h, the soft-agar layer was covered with 90 μl of the same culture medium without agar, and 10 μl of LP-184 or control medium were added and left on the cells for another 7 days (continuous exposure, 100 μl drug overlay). Compounds were serially diluted in DMSO, transferred in cell culture medium, and added to the assay plates by using a Tecan Freedom EVO 200 robotic platform. The DMSO concentration was kept constant at 0.3% v/v across the assay plate. Final concentrations of LP-184 were in the range of 1 nM – 10 μM. Every 96 well plate included six DMSO-treated control wells and drug-treated wells in duplicate at 9 concentrations. During incubation, cultures were monitored for colony growth using an inverted microscope. Within this period, *ex vivo* tumor growth led to the formation of colonies with a diameter of >50 μm (area >2000 μm^2^). At the time of maximum colony formation, vital colonies were stained for 48 h with a sterile aqueous solution of INT (2-(4-iodophenyl)-3-(4-nitrophenyl)-5-phenyltetrazolium chloride, 1 mg/mL, 25 μl/well), and colony counts were performed with an automatic image analysis system (CellInsight NXT, Thermo Scientific or Bioreader 5000 Vα, BIO-SYS). IC50 values were calculated as described above.

### Data analyses

Dose response curves associated with the data in Figure 1 were generated in GraphPad Prism version 9 using nonlinear regression-based curve fitting for inhibitor concentration versus normalized response. Pearson correlation coefficient indicated in Figure 2A was calculated using the Microsoft Excel function PEARSON. All box plots appearing in main and supplemental figures were produced using the R/ggplot2 software package. Associated statistics were run considering a significance level of 0.05 and two-tailed hypothesis testing, using the web tool provided in the link: https://www.socscistatistics.com/tests/studentttest/default2.aspx. The heatmap in Figure 4 was generated using R with the pheatmap package. R software was also used to plot PTGR1 expression and matching mutations for selected genes for each patient ID in Figure 6A.

Cell line gene expression data were obtained from the Cancer Cell Line Encyclopedia (CCLE) [41], mutation data from the Cancer Dependency Map portal (DepMap) [42]. Drug sensitivity data on approved drugs Oxaliplatin, Cisplatin, Pemetrexed, Paclitaxel, Gemcitabine were obtained from the Genomics of Drug Sensitivity in Cancer (GDSC) [43] database. Statistical two-tailed *t-test* analyses at a significance level of 0.05 were performed to evaluate any subgroup differences. Lung Adenocarcinoma (LUAD) clinical data were downloaded from The Cancer Genome Atlas (TCGA) via the Firebrowse data download portal (http://firebrowse.org/). The data was in the form of log2 quantile normalized RSEM gene expression values. From the data, tumor samples were extracted (https://docs.gdc.cancer.gov/Encyclopedia/pages/TCGA_Barcode/) as per the TCGA sample barcode ID. A total of 517 tumor samples were identified in the LUAD data. Based on the PTGR1 expression values, we grouped the samples into high, intermediate, and low expression groups. The first quartile and third quartile cutoffs of the entire 517 patient expression values covering all the genes were used to classify patients into different groups. Patients having PTGR1 expression < first quartile were classified as low expression group, while patients having PTGR1 expression > third quartile were classified as high expression group. Remaining samples were classified as intermediate PTGR1 expressing group. There were a total 184 patients having high level of PTGR1 and 333 patients with intermediate PTGR1 expression levels. None of the LUAD samples expressed low level of PTGR1 based on our classification approach. Using this strategy, patients with PTGR1 above a log2 expression value of 9.71 (333 of 517 patient samples or 64.4%) were considered as high expressors, between 2.92 and 9.71 as intermediate expressors (184 of 517 patient samples or 35.6%), and below 2.92 as low expressors (0 of 517 patient samples). Using the mutation data downloaded from UCSC Xena Browser (https://xenabrowser.net/) we identified mutations in selected genes. The 184 high PTGR1 expressing patients were queried for any of the deleterious mutations present in those genes.

### Gene expression and enrichment analysis

Pearson’s Correlation Coefficient (PCC) was calculated between all gene expression values and the z-score of LP-184 sensitivity, in all cell lines. Z-score was calculated by first taking the -log10 of the IC50 (M) values, and then scaling to produce final z-scores by subtracting the mean -log10 value and dividing by the standard deviation. The values with absolute PCC values greater than 0.5 were extracted and used separately for enrichment analysis. Enrichment was performed by calculating the overlap between the positive and negative PCC gene lists with pathways or gene sets in the target databases and evaluating the significance by Fisher’s exact test. For graphical visualization, *P* values from Fisher’s exact test were converted to -log10 *P* values. Wikipathways based gene pathways evaluated all pathways in the 2019 version of the Human pathways in the database (https://www.wikipathways.org). Kinase Perturbation enrichment was based on the libraries of Kinase Perturbations from GEO (up or down), which are gene set libraries from Gene Expression Omnibus (GEO) data (https://www.ncbi.nlm.nih.gov/geo/) annotated by the Ma’ayan lab (https://maayanlab.cloud/Enrichr/), using significantly differentially expressed genes in the up- or down-direction as a result of perturbations to kinases. Enrichment of transcriptional regulators used a similar gene-set library that was instead developed from perturbations to transcription factors, also constructed by the Ma’ayan lab based on GEO data of transcription-factor perturbations.

### Tumor xenograft study in mice

The dosing solutions of LP-184 were freshly prepared from powder material by dissolving in EtOH then adding saline (final concentration being 5% EtOH and 95% saline). NCI-H460 cells were acquired from the ATCC (ATCC, HTB-177) and cultured in RPMI + L-Glutamine, supplemented with 10% FBS, and 1% Penicillin/Streptomycin solution, in a humidified incubator at 37°C and 5% CO_2_. Cells were split at 80% confluency and harvested for implantation at 70% confluency. Athymic Nude mice were procured through Jackson Laboratory (Strain 002019, 5 weeks old; all from isolator room RB06). Mice were fed Teklad irradiated (sterilized) mouse diet and bedded with Teklad irradiated (sterilized) corncob bedding from Envigo (Indianapolis, IN). Mice were housed in Optimice carousel sterile quarters with filtered air supply in disposable cages from Animal Care Systems, Inc. (Centennial, CO, USA). On the day of implantation, NCI-H460 cells were trypsinized and allowed to detach from flasks. Trypsin was then neutralized with complete media and cells were spun at 400 × g. Media was aspirated and cells were washed with phosphorous buffered saline (PBS) without Ca^2+^ or Mg^2+^. Cells were resuspended in RPMI at a concentration of 2 × 10^7^ cells/mL. An equal volume of Matrigel (Lot # 1552475) was added for a final concentration of 1 × 10^7^ cells/mL. A volume of 100 μL was injected subcutaneously into the right hind flank of each animal using a 27g needle (a total of 1 × 10^6^ cells). Tumors were measured with a digital caliper for the duration of the study. Tumors were measured in two dimensions using calipers, and volume was calculated using the formula: Tumor Volume (mm^3^) = w^2^ × l/2, where *w* = width and *l* = length, in mm, of the tumor. For this study, the calipers were aligned to the tumor edges (the tumors were not squeezed with the caliper). Resulting tumors were monitored by calipering twice weekly. Animal weights were measured twice weekly. Animal behavior was monitored daily. All mice were maintained in isolated housing at constant temperature and humidity. Treatment was started after three weeks when the tumors reached an average volume of 150 mm^3^. Animals were randomly divided into 2 groups (*n* = 10 in each group) and administered intraperitoneally with (a) 5% ethanol and 95% saline as vehicle for the control group and (b) 5 mg/kg LP-184 in vehicle for the treatment group, in 4 doses on days 1, 3, 6, 9 and 12. Tumor volumes and body weights were measured on days 1, 5, 8, 12 and 15. Statistical analysis was performed using GraphPad Prism version 9. Data were processed for Two-Way ANOVA using Geisser-Greenhouse correction and Sidak’s post hoc analysis for group comparisons.

## SUPPLEMENTARY MATERIALS



## Abbreviations

NSCLC: Non-small Cell Lung Cancer
PTGR1: Prostaglandin Reductase 1
TC-NER: Transcription Coupled-Nucleotide Excision Repair
PCC: Pearson’s Correlation Coefficient
CCLE: Cancer Cell Line Encyclopedia
GDSC: Genomics of Drug Sensitivity in Cancer
LUAD: Lung Adenocarcinoma
TCGA: The Cancer Genome Atlas
