# Supplementary material for: The acylfulvene alkylating agent, LP-184, retains nanomolar potency in non-small cell lung cancer carrying otherwise therapy-refractory mutations
Source: Oncotarget. 2021 Apr 13;12(8):791–806. doi: 10.18632/oncotarget.27943 (PMC8057270; doi:10.18632/oncotarget.27943)
Supplement: Supplementary file 1 [file oncotarget-12-791-s001.pdf]

The acylfulvene alkylating agent, LP-184, retains nanomolar potency in non-small cell lung cancer carrying otherwise therapy-refractory mutations

SUPPLEMENTARY MATERIALS

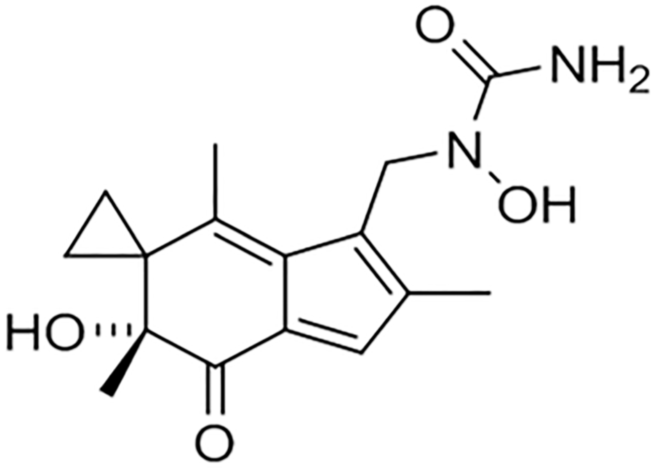

Supplementary Figure 1: LP-184 chemical structure.

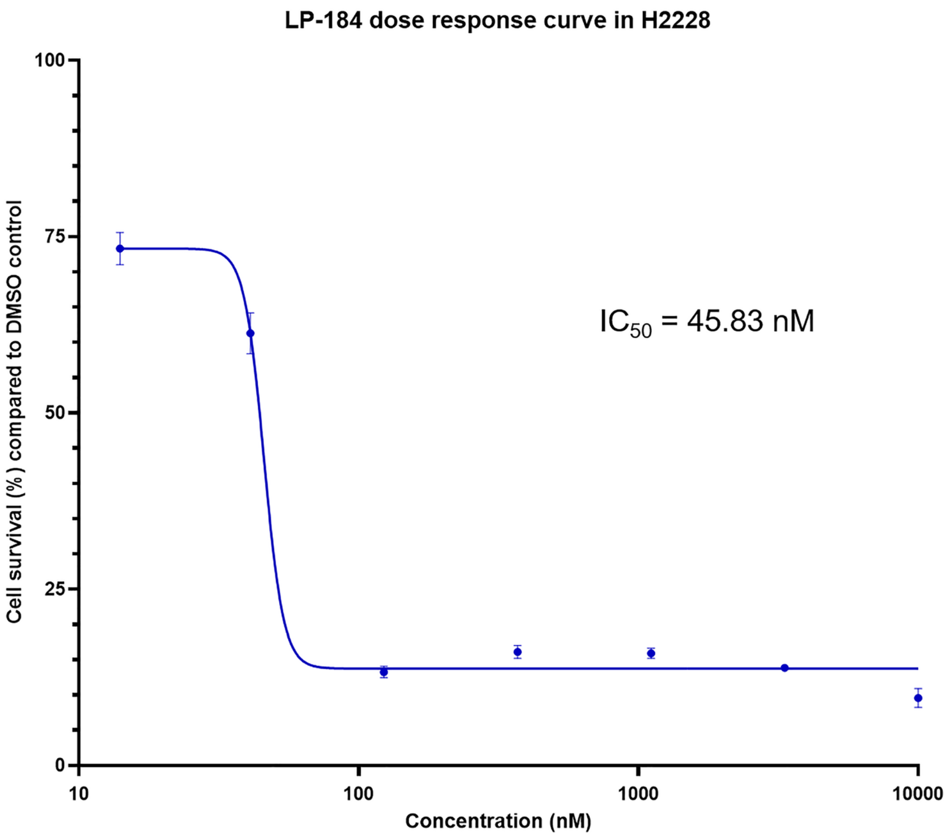

Supplementary Figure 2: LP-184 dose response curve in H2228.

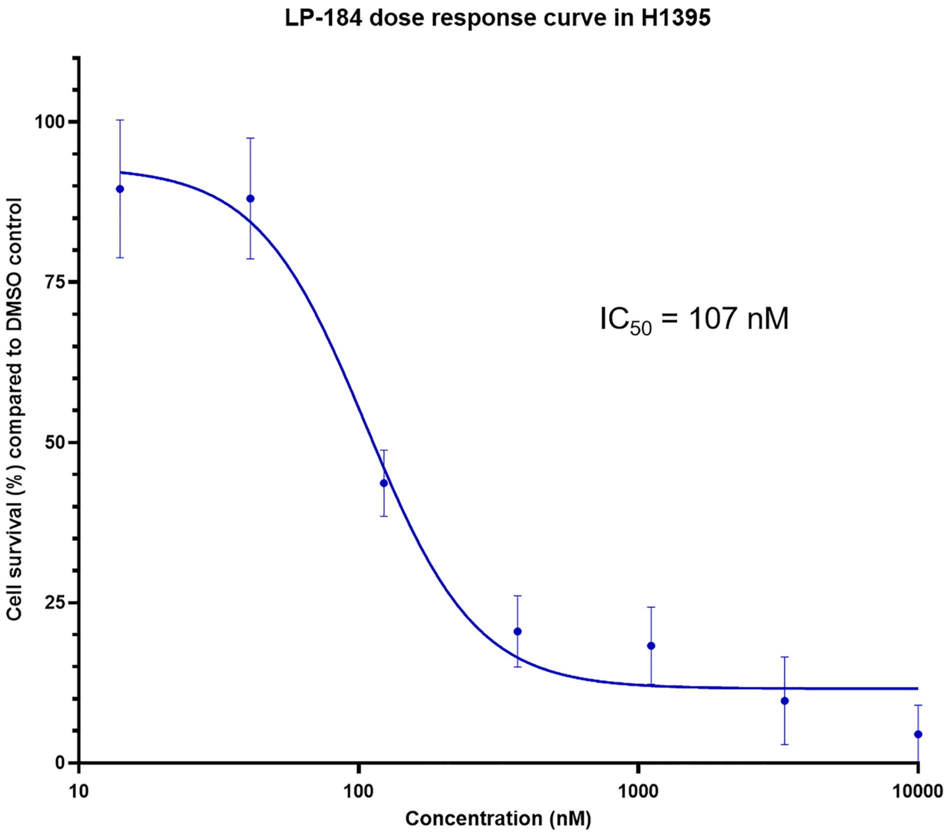

Supplementary Figure 3: LP-184 dose response curve in H1395.

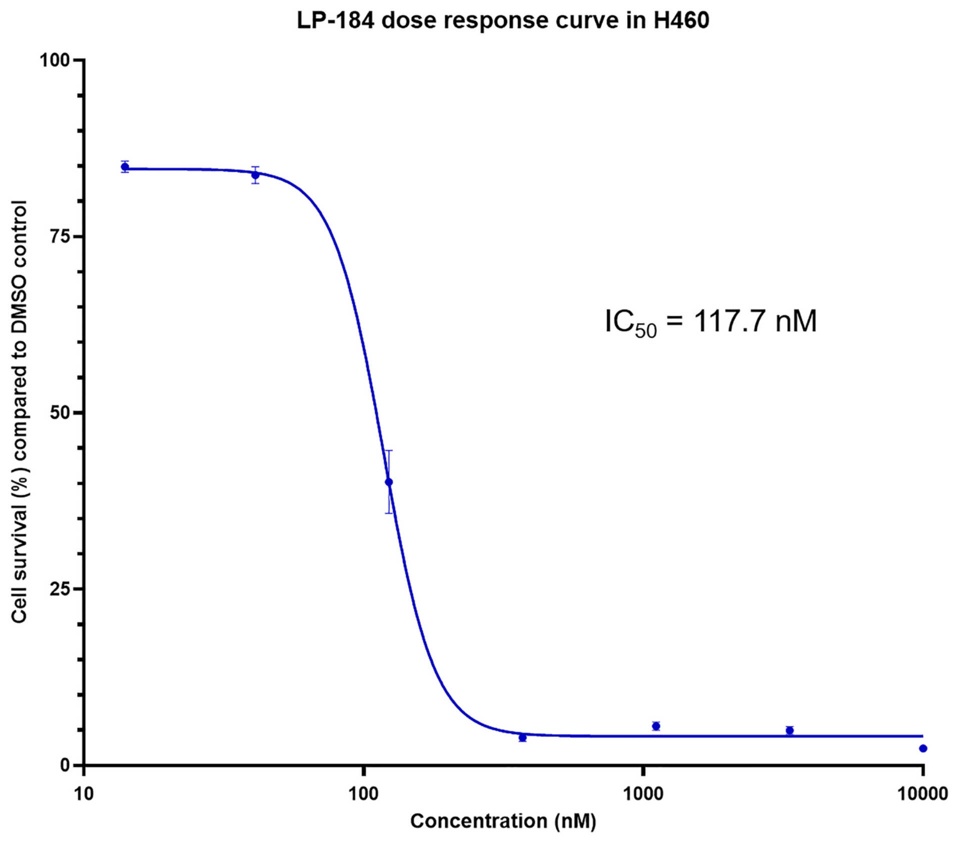

Supplementary Figure 4: LP-184 dose response curve in H460.

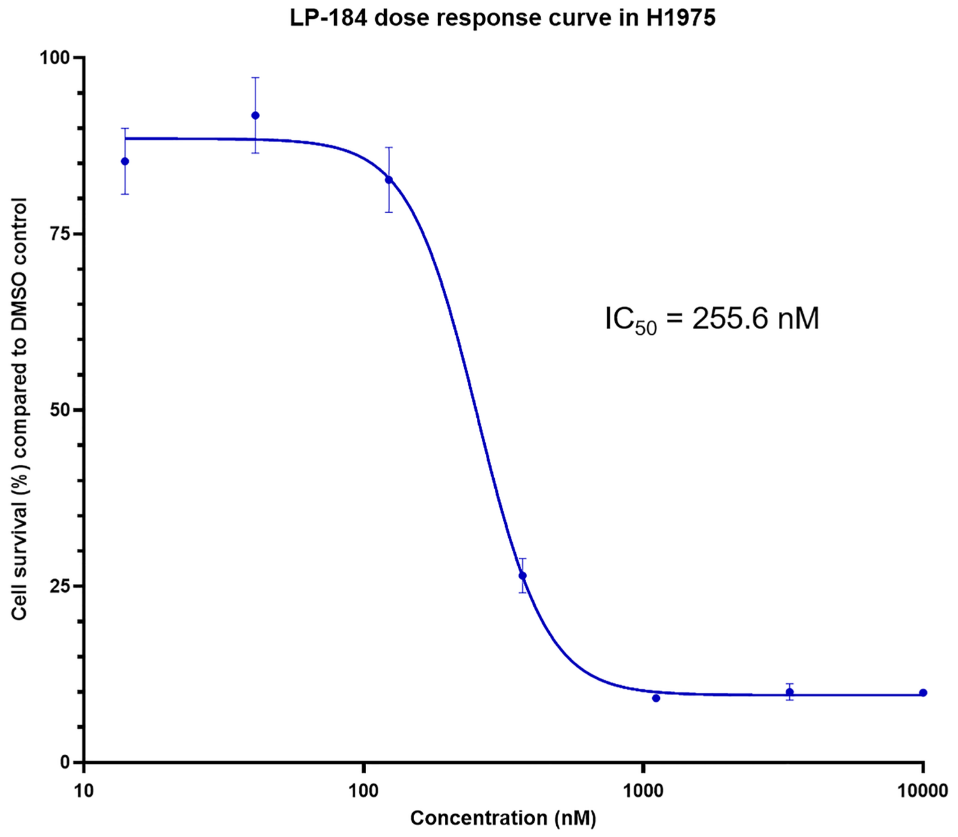

Supplementary Figure 5: LP-184 dose response curve in H1975.

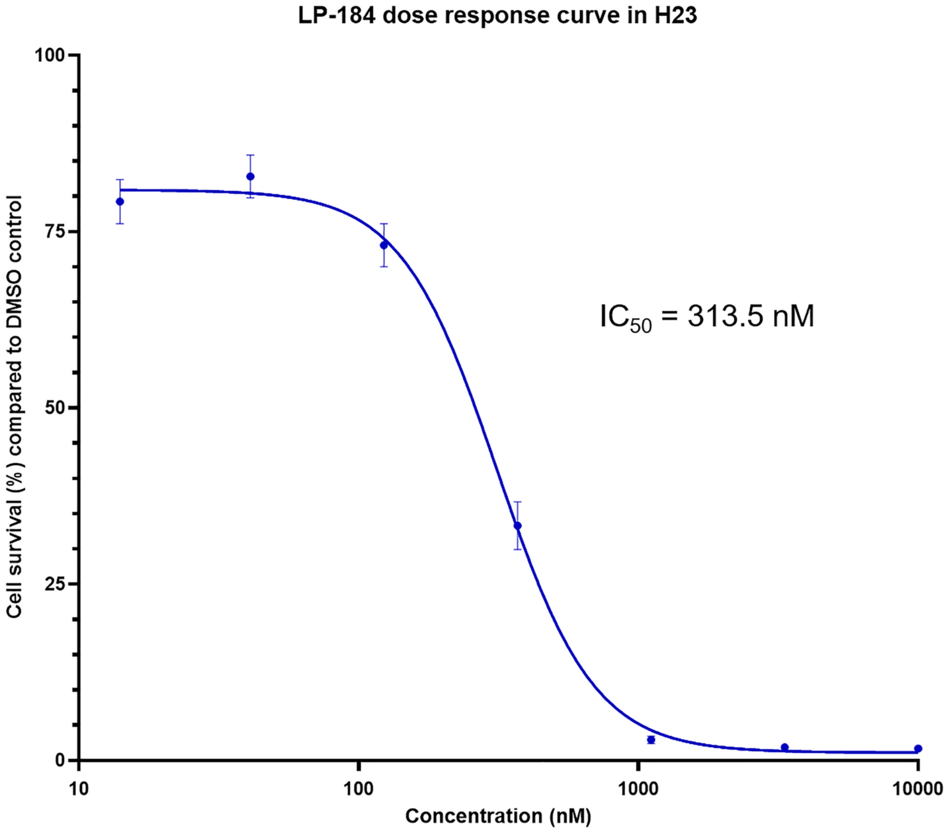

Supplementary Figure 6: LP-184 dose response curve in H23.

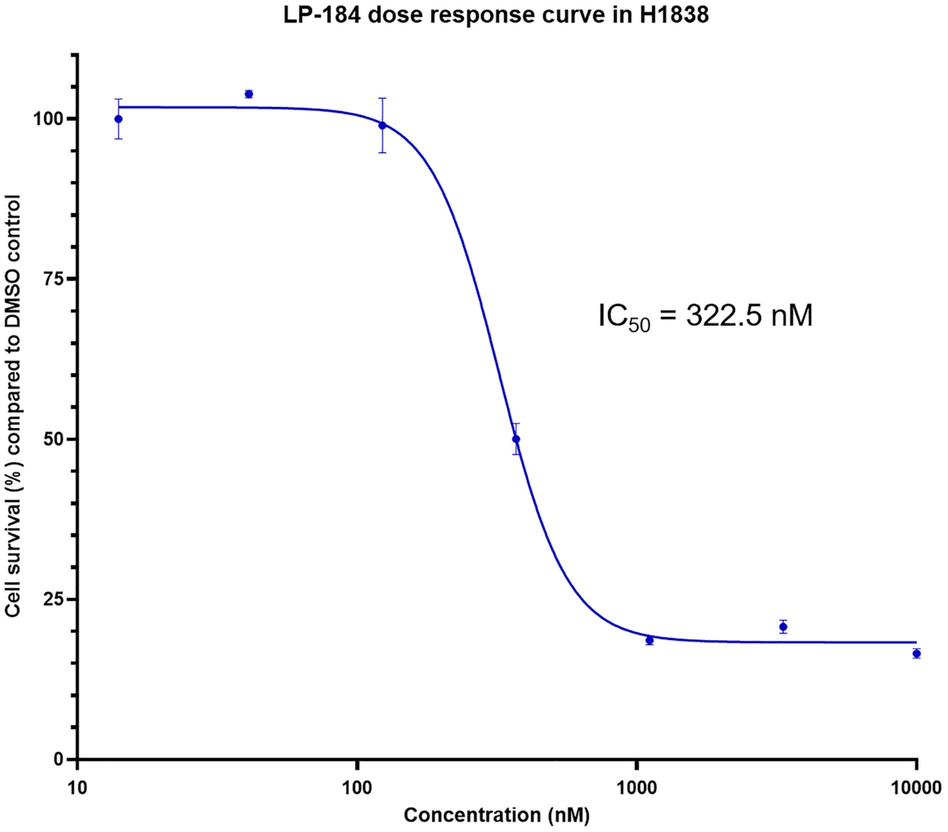

Supplementary Figure 7: LP-184 dose response curve in H1838.

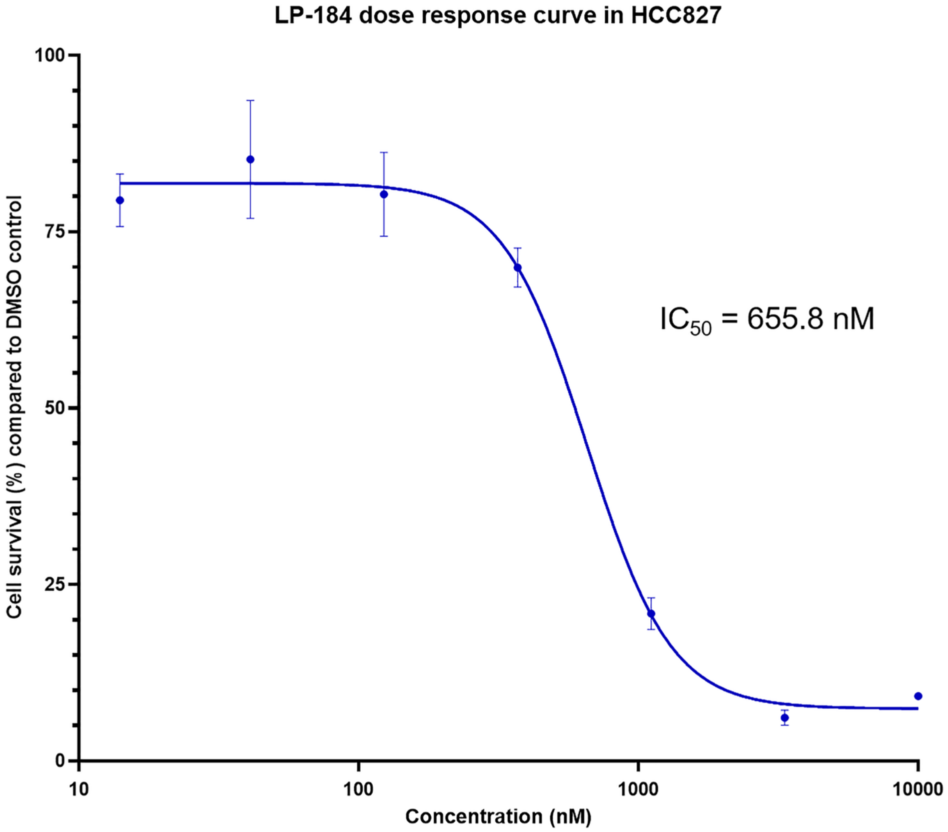

Supplementary Figure 8: LP-184 dose response curve in HCC827.

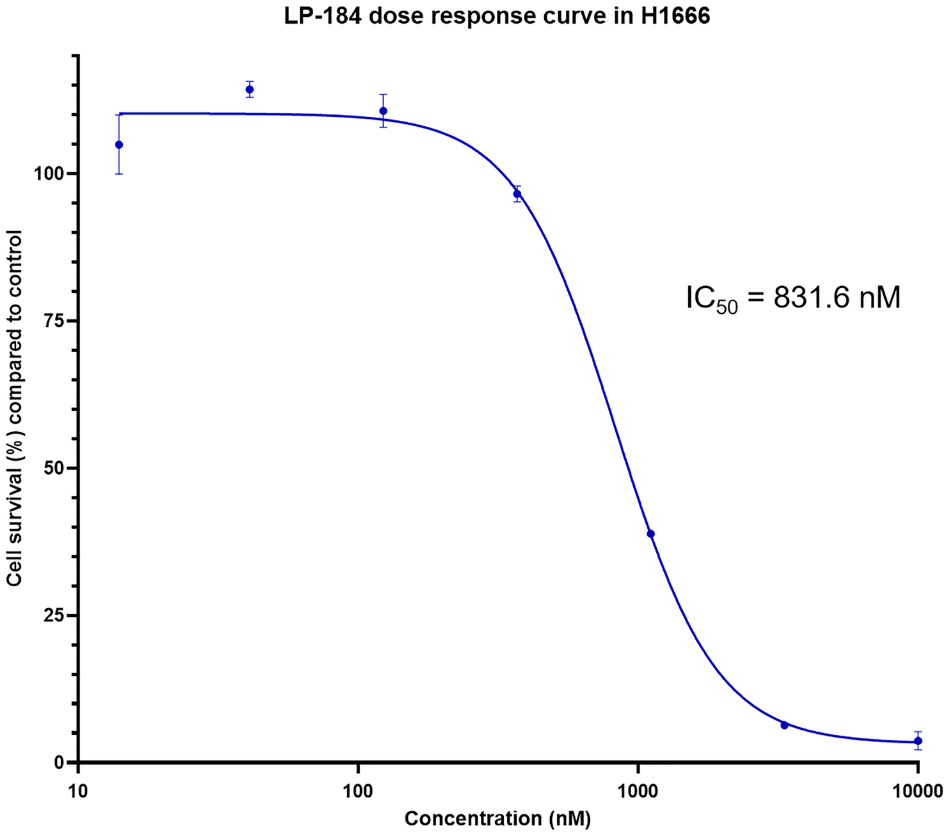

Supplementary Figure 9: LP-184 dose response curve in H1666.

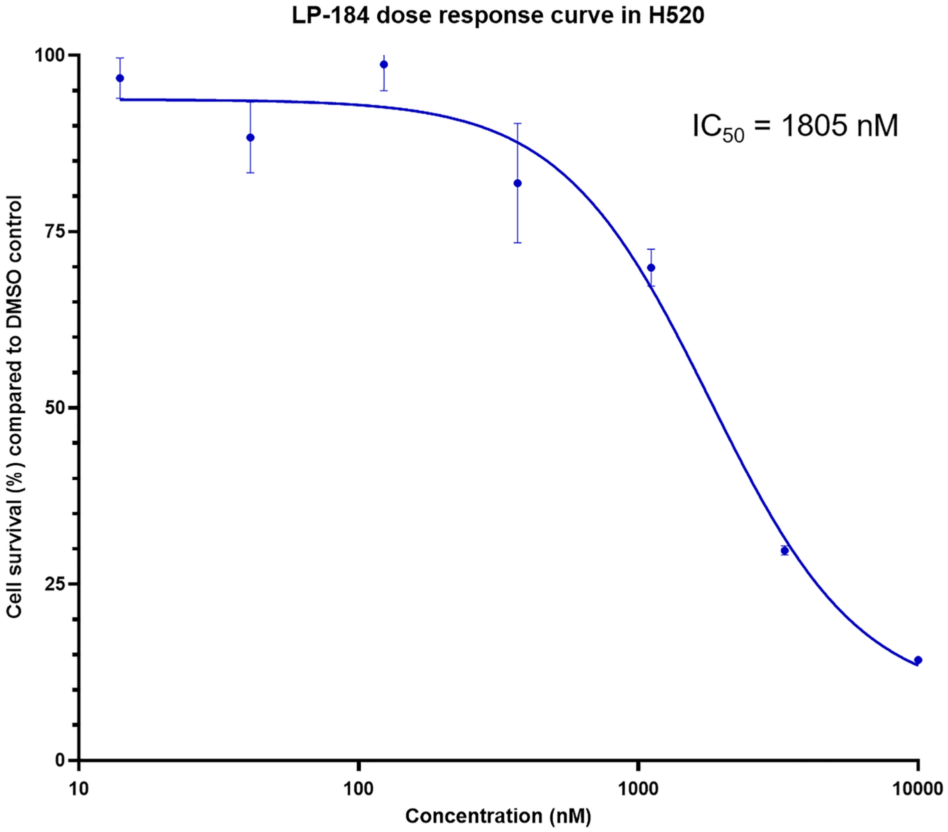

Supplementary Figure 10: LP-184 dose response curve in H520.

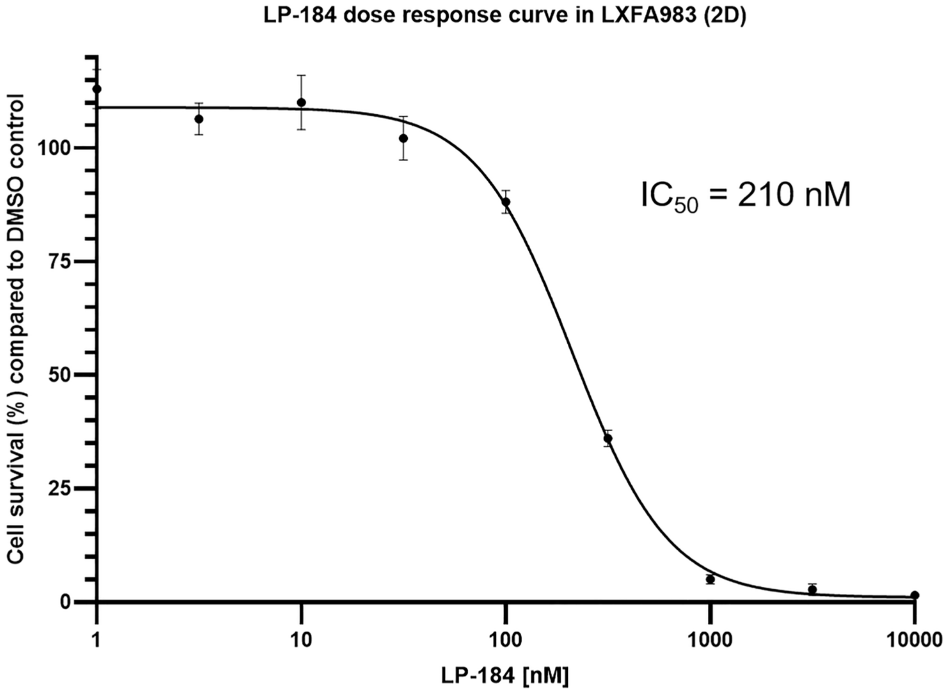

Supplementary Figure 11: LP-184 dose response curve in LXFA983(2D).

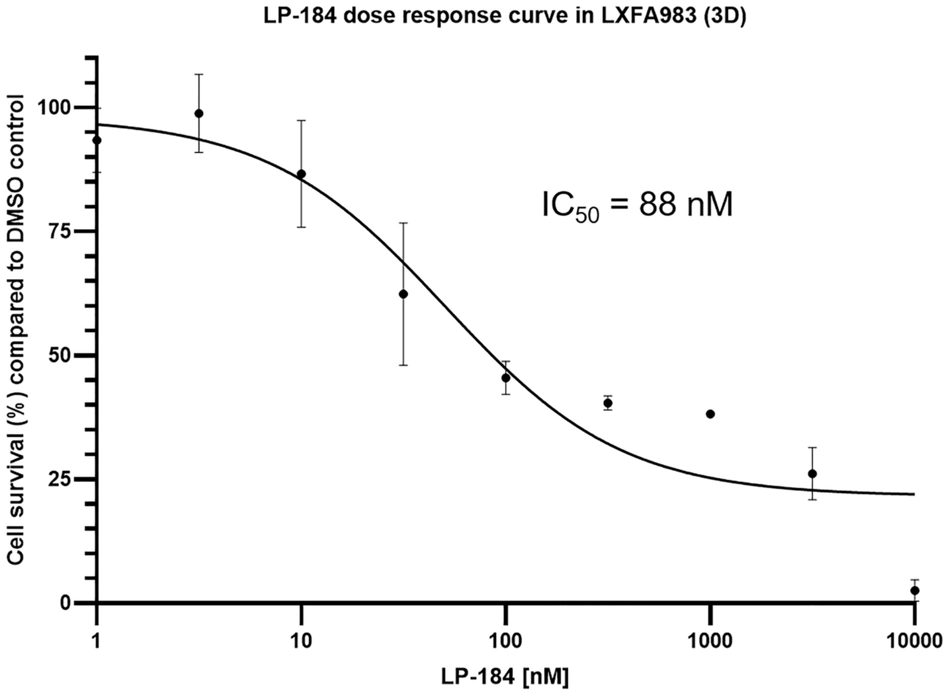

Supplementary Figure 12: LP-184 dose response curve in LXFA983(3D).

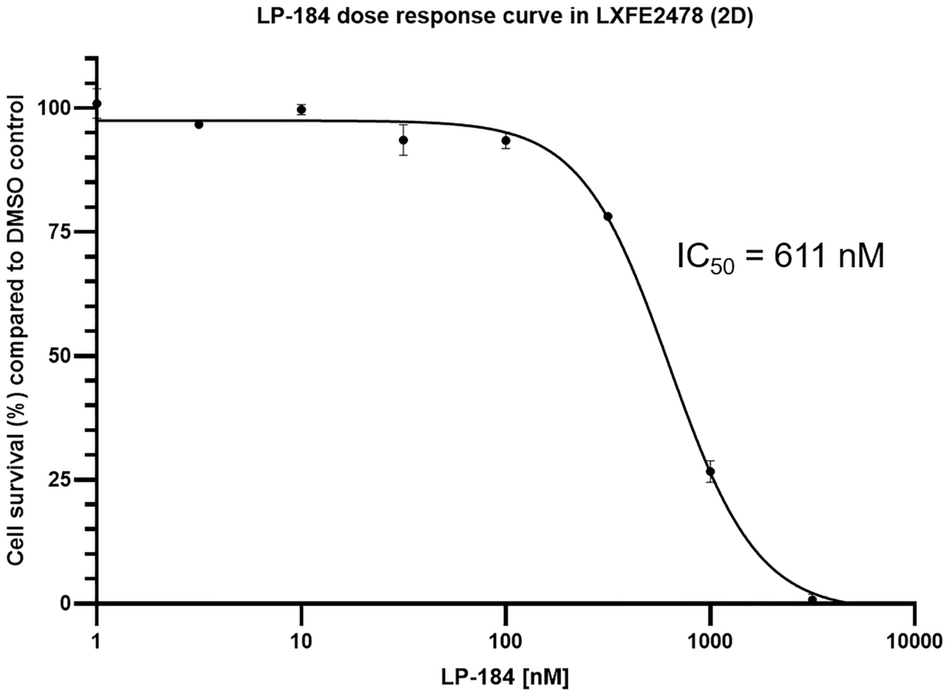

Supplementary Figure 13: LP-184 dose response curve in LXFE2478(2D).

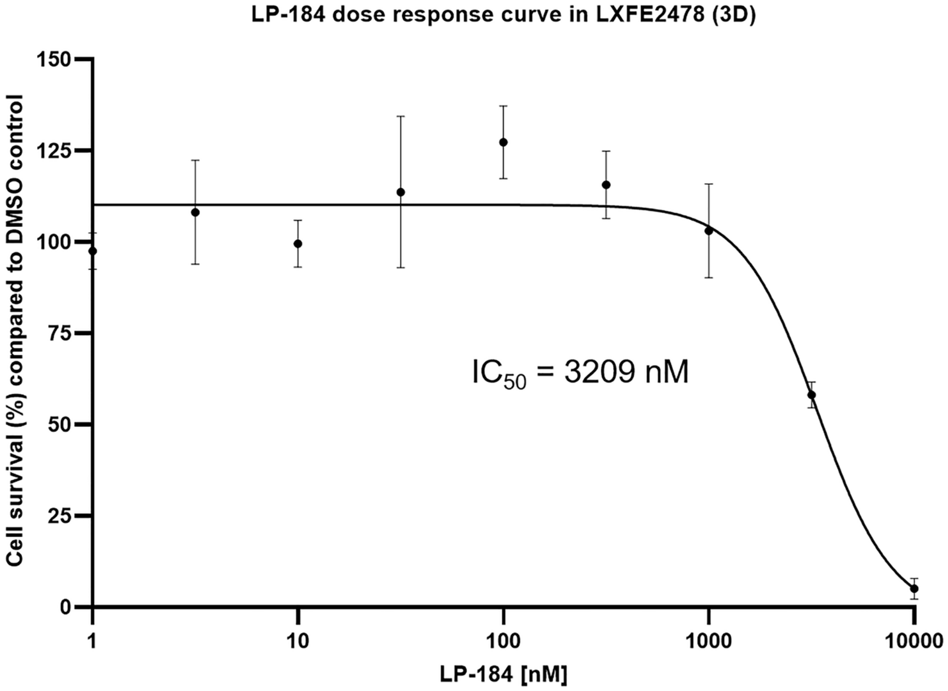

Supplementary Figure 14: LP-184 dose response curve in LXFE2478(3D).

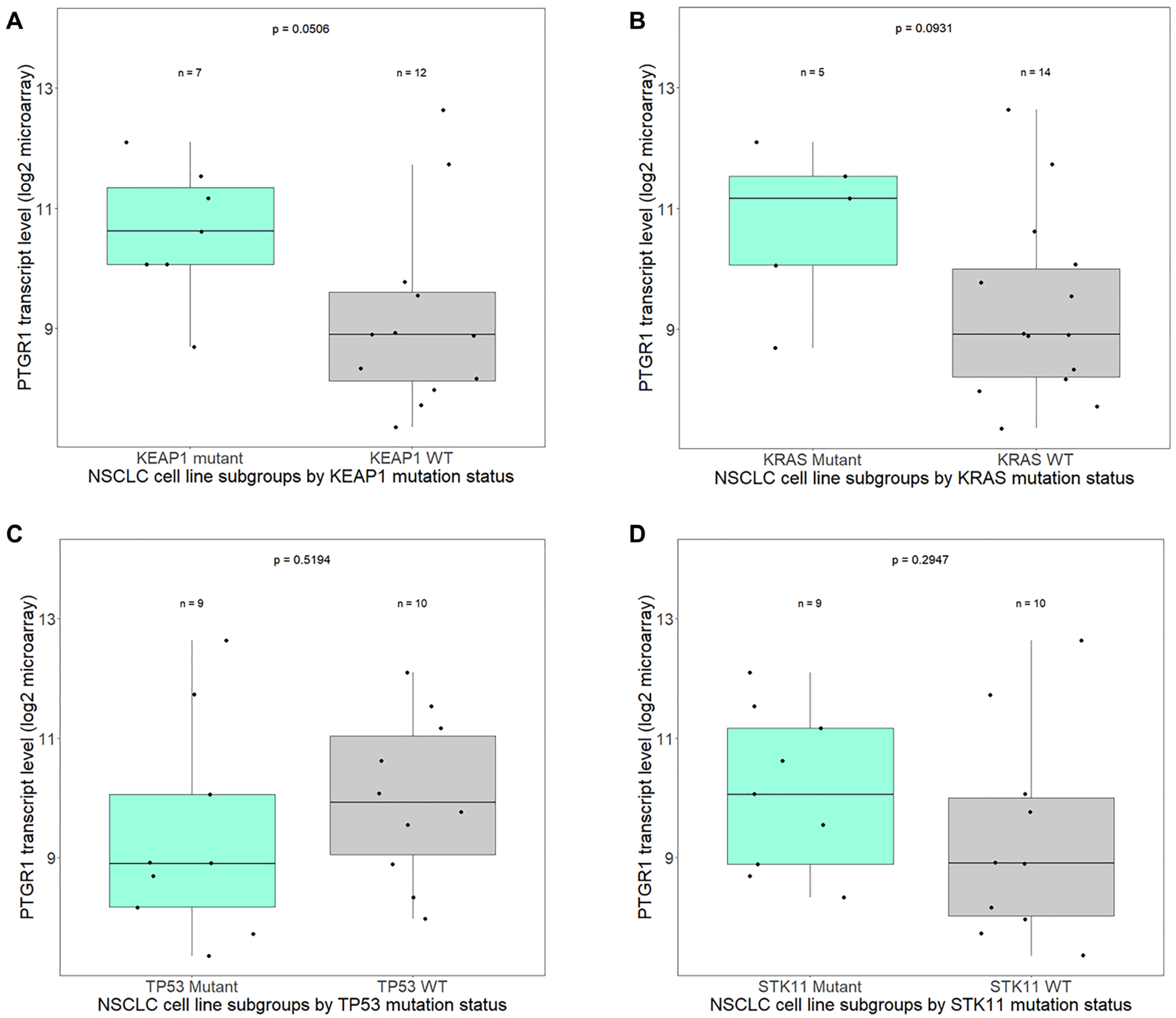

Supplementary Figure 15: PTGR1 transcript levels in NSCLC cell lines are largely not influenced by mutation status of key oncogenes and tumor suppressors.

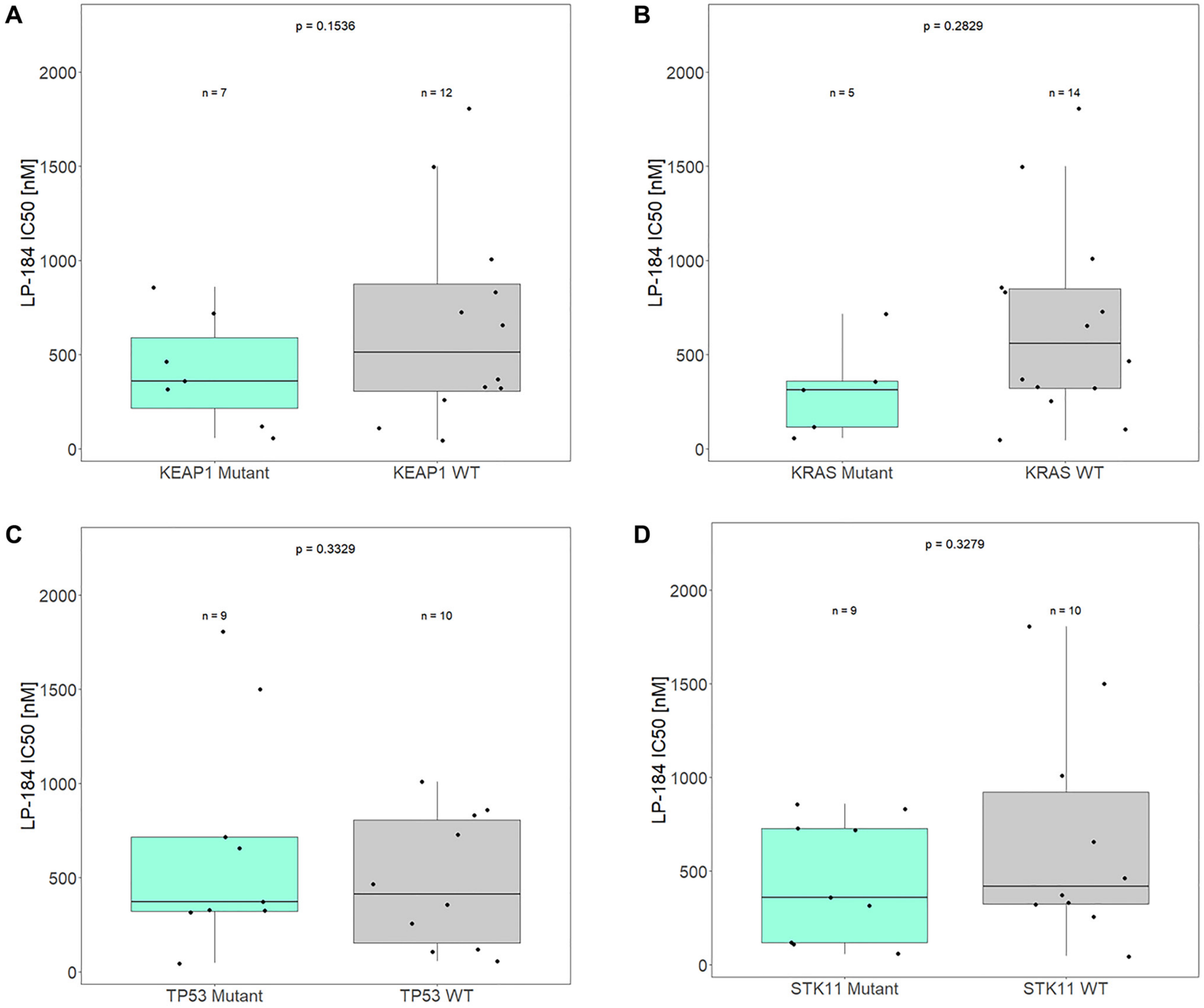

Supplementary Figure 16: LP-184 sensitivity in cell lines is independent of mutations in key oncogenes and tumor suppressors. Box plots statistically compare LP-184 sensitivities between cell line groups that are either wild type or mutant for (**A**) KEAP1 (**B**) KRAS (**C**) TP53 and (**D**) STK11.

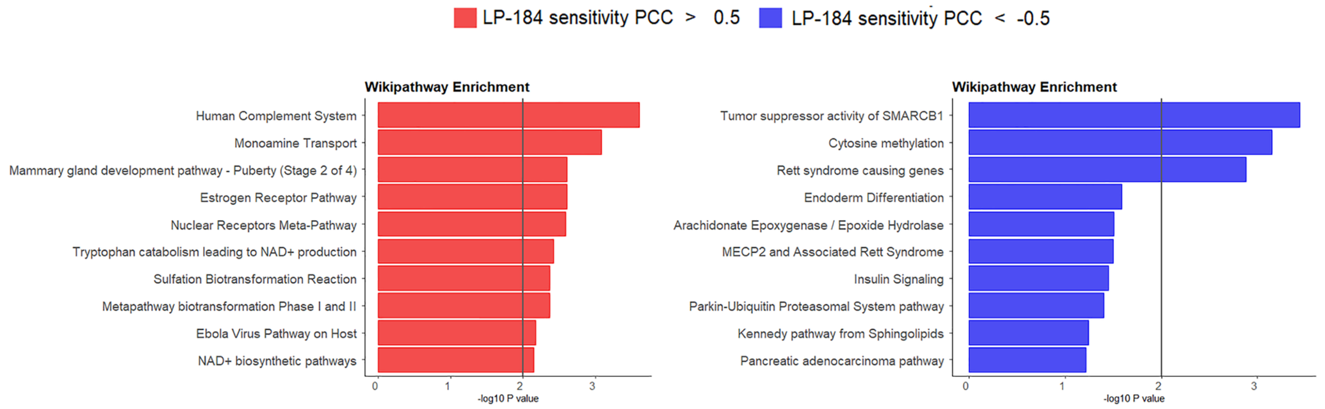

Supplementary Figure 17: Wikipathway gene enrichment of genes highly correlated with LP-184 sensitivity, in the positive (red) or negative (blue) direction. *P* values were calculated by Fisher's exact test and shown with bar charts as -log10 transformations.

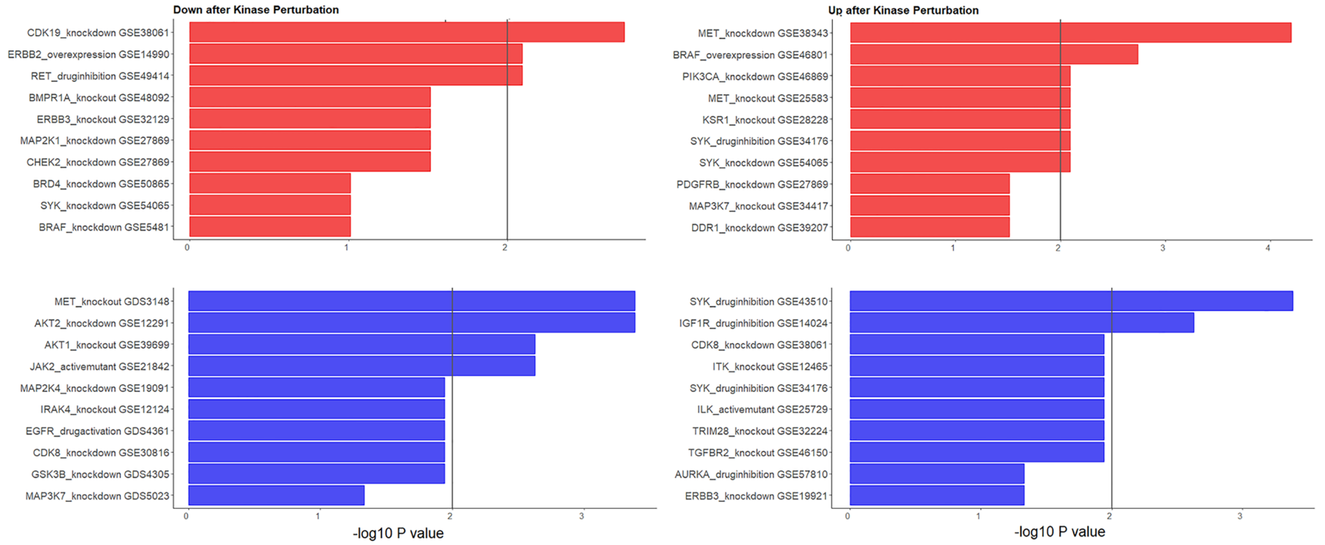

Supplementary Figure 18: Relationship between LP-184 correlated genes and kinase signaling pathways. Enrichment performed on the same gene list as in (A), but libraries were composed of genes differentially expressed following kinase gain- or loss-of-function perturbations.

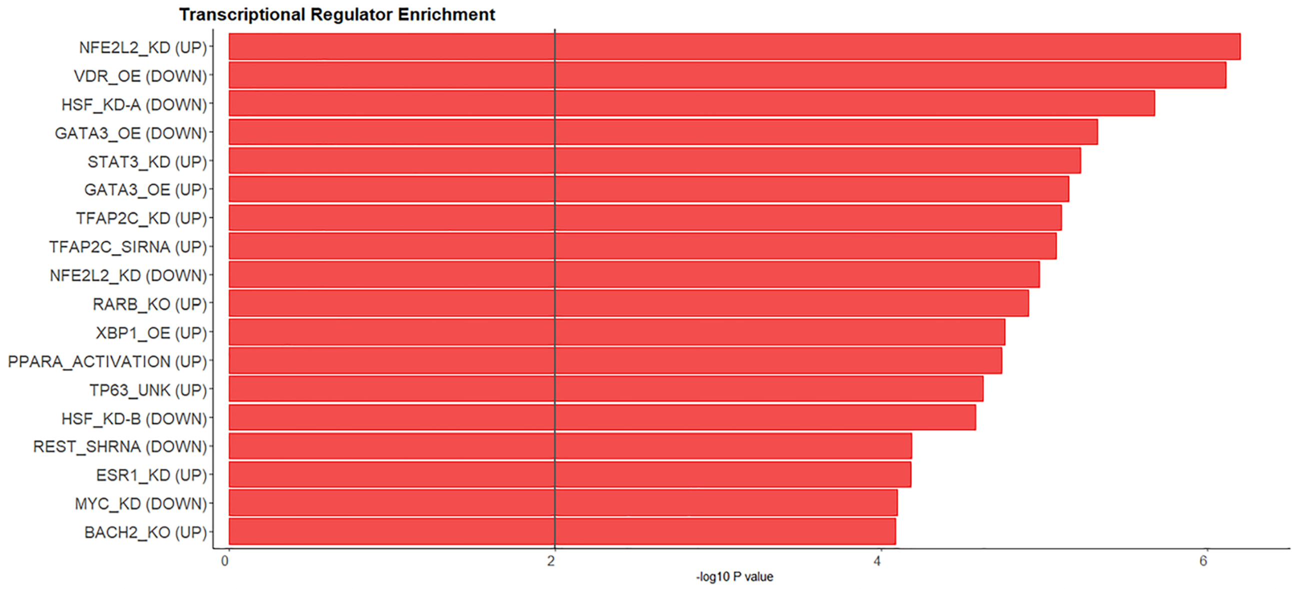

Supplementary Figure 19: LP-184 response genes associated with transcriptional regulators. Enrichment performed on the same gene list as in (A), but libraries were composed of genes differentially expressed following transcription factor perturbations.

Supplementary Table 1: NSCLC cell lines used and their growth conditions

	Cell Line	Base Medium	% of FBS	Freezing Media	Subcultivation
**1**	HCC827	RPMI-1640	10%	growth medium + 5%DMSO	1:4 to 1:6
**2**	H1975	RPMI-1640	10%	growth medium + 5%DMSO	1:3 to 1:6
**3**	H2228	RPMI-1640	10%	growth medium + 5%DMSO	1:2 to 1:3
**4**	H23	RPMI-1640	10%	growth medium + 5%DMSO	1:3 to 1:6
**5**	H460	RPMI-1640	10%	growth medium + 5%DMSO	1:3 to 1:8
**6**	H520	RPMI-1640	10%	growth medium + 5%DMSO	1:3 to 1:6
**7**	H1944	RPMI-1640	10%	growth medium + 5%DMSO	1:3 to 1:6
**8**	H1395	RPMI-1640	10%	growth medium + 5%DMSO	1:3 to 1:6
**9**	H1838	RPMI-1640	10%	growth medium + 5%DMSO	1:3 to 1:4
**10**	H1703	RPMI-1640	10%	growth medium + 5%DMSO	1:3 to 1:6
**11**	H1563	RPMI-1640	10%	growth medium + 5%DMSO	1:3 to 1:4
**12**	H1573	RPMI-1640	5%	growth medium + 7.5%DMSO	1:2 to 1:3
**13**	H1435	RPMI-1640	5%	85%RPMI + 10%FBS + 5%DMSO	1:3 to 1:6
**14**	H1648	RPMI-1640	5%	growth medium + 5%DMSO	1:3 to 1:6
**15**	H1666	RPMI-1640	5%	85%RPMI + 10%FBS + 5%DMSO	1:2 to 1:4
**16**	A549	F-12K	10%	growth medium + 5%DMSO	1:3 to 1:8
**17**	H1793	DMEM:F12	5%	85%DMEM/F12 + 10%FBS + 5%DMSO	1:3 to 1:6
**18**	LC-2/ad	RPMI-1640:F12	10%	growth medium + 5%DMSO	1:2 to 1:4
**19**	HCC 78	RPMI-1640	10%	growth medium (70%), FBS (20%), DMSO (10%)	1:2

Supplementary Table 2: NSCLC cell lines tested and their characteristics

Oncogene/tumor suppressor mutation status					Clinical annotation		
**Cell line**	**KRAS**	**KEAP1**	**TP53**	**STK11**	**Gender**	**Smoking history**	**NSCLC histology**
HCC827	WT	WT	V218 in-frame deletion	WT	Female	Unknown	Adenocarcinoma
H1975	WT	WT	WT	WT	Female	Non-smoker	Adenocarcinoma
H2228	WT	WT	Q331X*	WT	Female	Non-smoker	Adenocarcinoma
HCC78	WT	WT	S241F	WT	Male	Unknown	Adenocarcinoma
LC-2/ad	NA	WT	Unknown	WT	Female	Unknown	Adenocarcinoma
A549	G12S	G333C	WT	p.Q37*	Male	Unknown	Adenocarcinoma
H23	G12C	Q193H	M153I	p.W332*	Male	Unknown	Adenocarcinoma
H460	Q61H	D236H	WT	p.Q37*	Male	Unknown	Large cell lung carcinoma
H1435	WT	R413L	WT	p.S299F	Female	Non-smoker	Adenocarcinoma
H1648	WT	G364C, G430C	WT	WT	Male	Smoker	Adenocarcinoma
H520	WT	WT	W146X	WT	Male	Unknown	Squamous Cell Carcinoma
H1573	G12A	A143P	R248L	p.S216F	Female	Smoker	Adenocarcinoma
H1793	WT	WT	R209X	WT	Female	Non-smoker	Adenocarcinoma
H1944	G13D	R272L	WT	p.K62N, K78N	Female	Smoker	Adenocarcinoma
H1395	WT	WT	WT	p.L55fs	Female	Smoker	Adenocarcinoma
H1838	WT	WT	R273L	WT	Female	Non-smoker	Adenocarcinoma
H1703	WT	WT	WT	WT	Male	Smoker	Squamous Cell Carcinoma
H1563	WT	WT	WT	p.G242W	Male	Non-smoker	Adenocarcinoma
H1666	WT	WT	WT	p.K235fs	Female	Non-smoker	Adenocarcinoma

Supplementary Table 3A: LP-184 IC50 data across different primary NSCLC cell lines used in this study

NSCLC cell line	LP-184 IC50 [nM]
H2228	45.83
H1944	55.99
H1395	107
H460	117.7
H1975	255.6
H23	313.5
H1838	322.5
H1793	328.5
A549	358.9
LC-2/ad	371
H1648	464.5
HCC827	655.8
H1573	716.5
H1563	727.3
H1666	831.6
H1435	859.1
H1703	1009
HCC78	1499
H520	1805

Supplementary Table 3B: LP-184 IC50 data across different brain metastasis models from primary NSCLC used in this study

Brain metastasis cell line	LP-184 IC50 [nM]
LXFA983 (2D, primary lung)	210
LXFE2478 (2D, primary lung)	611
LXFA983 (3D, primary lung)	88
LXFE2478 (3D, primary lung)	3209

Supplementary Table 4: IC50 data for approved drugs in selected NSCLC cell lines, obtained from the GDSC database

	IC50 [nM]				
**NSCLC Cell Line**	**Oxaliplatin**	**Cisplatin**	**Pemetrexed**	**Paclitaxel**	**Gemcitabine**
H2228	93243.08	18301	39405	188	433.182
H1944	47390.532	79386.377	24282	1378	136.49
H1975	64369.499	244733.098	40570	47	493.797
H23	36268.15	7143.533	15984	403	50.644
H1838	334366.881	477403.948	112010	4403	121240.315
H1793	250239.641	1252986.995	182960	2486	7099.818
H1648	5945.864	31453.527	69434	8	18034.068
HCC827	60609.886	96136	28930	876	13999.247
H1573	246195.481	470856.804	72372	1309	316283.811
H1563	228445.286	379156.283	78578	1328	150020.452
H1666	56355.149	509337.922	40363	1023	1575.582
H1435	210403.18	98435.644	58806	596	11751.627
H1703	42003.033	206242.091	56674	11	10.093
HCC78	60279.719	70162	5558	351	11.112
H520	34630.235	17796.818	8199	76	79.883

Supplementary Table 5: IC50 data for LP-184 in other comparator cell lines

Non-target comparator cell line (tissue type)	LP-184 IC50 [nM]
CHRF 288-11 (megakaryocyte)	8,800 (9)
K562 (chronic myeloid leukemia)	>10,000
8392 (B lymphocyte)	>100,000

Supplementary Table 6: Tumor size in mm^3 by group

Group	Mouse	Tumor Volume (mm^3) on Sampling Day				
		**1**	**5**	**8**	**12**	**15**
**1. Vehicle control**	**1**	380.70	951.67	1662.65	2588.60	3706.28
	**2**	340.78	737.33	1096.85	1903.88	3054.58
	3	208.74	392.67	492.72	650.25	905.91
	**4**	164.71	217.33	428.97	589.82	746.30
	**5**	111.25	314.41	579.78	794.98	1095.12
	**6**	101.12	210.70	462.72	749.91	1372.88
	**7**	221.39	303.75	576.00	801.43	784.16
	**8**	157.12	213.56	530.00	792.76	1085.94
	**9**	119.16	440.90	1169.62	2086.97	3072.00
	**10**	56.45	145.41	269.00	432.96	551.41
**2. LP-184 (5 mg/kg)**	**1**	437.40	477.14	423.77	348.17	437.38
	**2**	275.70	115.18	149.68	101.12	209.48
	**3**	194.48	133.10	93.29	53.24	87.26
	**4**	122.30	132.88	116.46	91.29	136.80
	**5**	109.65	97.47	70.23	40.57	59.90
	**6**	106.64	117.62	41.60	18.43	23.12
	**7**	227.25	397.81	257.91	115.34	229.15
	**8**	148.84	307.24	129.51	73.73	107.65
	**9**	102.85	150.70	112.09	71.79	127.01
	**10**	95.85	163.85	106.17	111.60	198.45

Supplementary Table 7: Mouse body weight in grams by group

Group	Mouse	Body Weight (grams) on Sampling Day				
		1	5	8	12	15
1. Vehicle control	1	23.3	23.6	23.4	24.3	24.2
	2	21.5	21.4	20.6	22.0	22.5
	3	24.9	24.8	24.2	24.9	25.0
	4	24.1	23.9	22.4	23.0	23.8
	5	24.5	24.6	24.5	25.1	25.0
	6	23.3	22.2	21.0	22.3	22.7
	7	22.8	22.2	22.7	22.9	22.8
	8	21.9	20.4	20.9	21.2	20.9
	9	23.3	21.7	23.1	23.5	24.3
	10	24.0	22.5	23.4	23.1	22.9
2. LP-184 (5 mg/kg)	1	22.6	21.1	18.5	16.5	14.5
	2	18.3	16.7	15.3	16.3	16.5
	3	22.3	20.4	16.9	14.8	16.4
	4	24.3	22.8	20.1	18.2	19.4
	5	25.4	23.5	21.4	20.6	22.4
	6	22.5	21.2	18.8	16.9	18.1
	7	21.5	19.9	20.3	18.8	19.4
	8	27.0	25.3	25.5	23.5	24.6
	9	26.1	24.1	24.9	23.9	24.2
	10	22.8	21.1	21.8	21.3	21.6

Supplementary Table 8: Tumor volume data in Vehicle control vs. LP-184 at 5 mg/kg were processed for Two-Way ANOVA using Geisser-Greenhouse correction and Sidak's post hoc analysis for group comparisons

Day	Mean difference in tumor volume (mm^3)	Adjusted *p* value
1	4.05	>0.9999
5	183.5	0.2957
8	576.8	0.0112
12	1037	0.0091
15	1476	0.0153
